# Supplementary figures and images for: Making information and communications technologies (ICTs) work for health: protocol for a mixed-methods study exploring processes for institutionalising geo-referenced health information systems to strengthen maternal neonatal and child health (MNCH) service planning, referral and oversight in urban Bangladesh
Source: BMJ Open. 2020 Dec 2;10(12):e032820. doi: 10.1136/bmjopen-2019-032820 (PMC7712401; doi:10.1136/bmjopen-2019-032820)

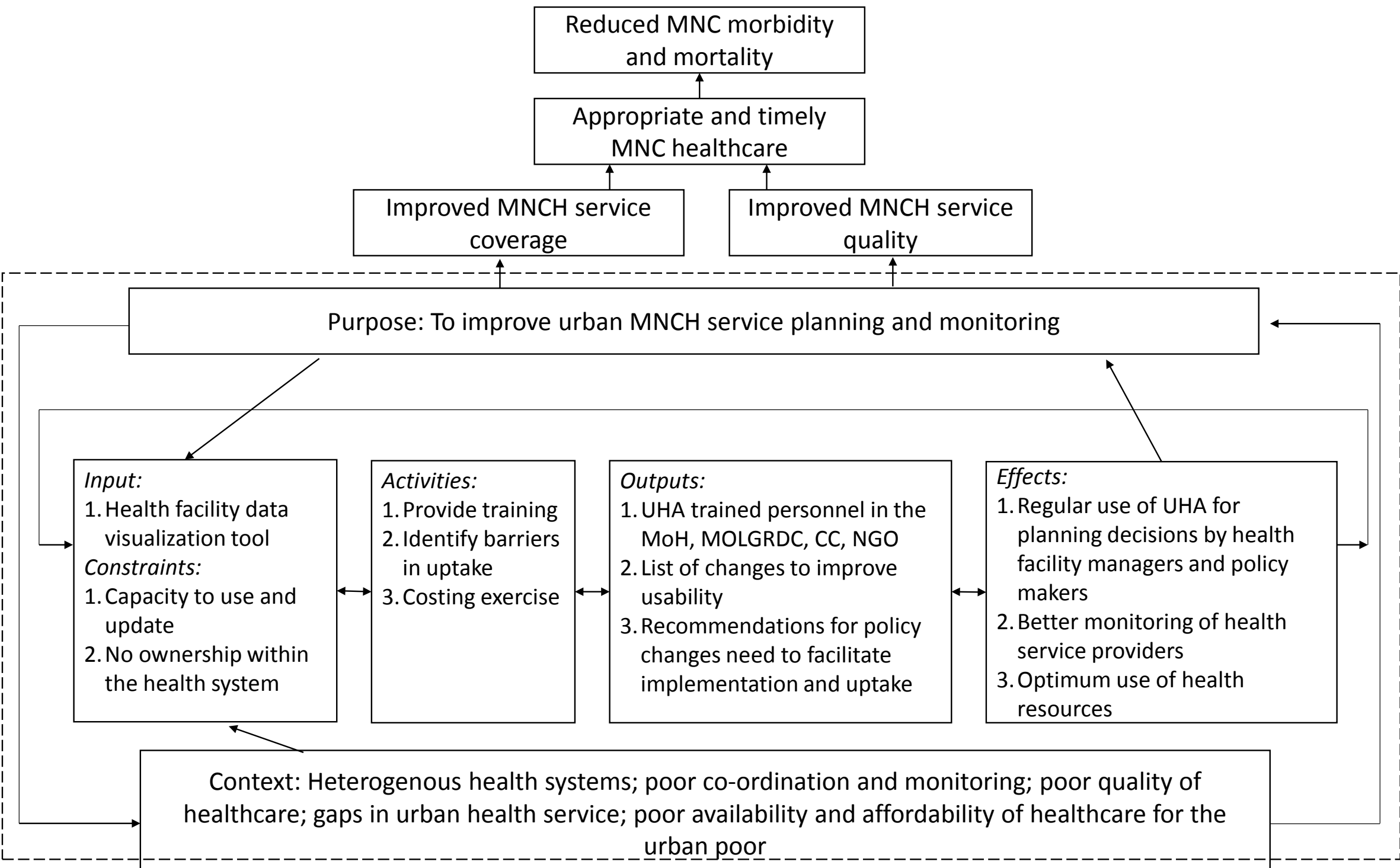

Supplement: Supplementary data [file bmjopen-2019-032820supp001.pdf]
