# Supplementary material for: Making information and communications technologies (ICTs) work for health: protocol for a mixed-methods study exploring processes for institutionalising geo-referenced health information systems to strengthen maternal neonatal and child health (MNCH) service planning, referral and oversight in urban Bangladesh
Source: BMJ Open. 2020 Dec 2;10(12):e032820. doi: 10.1136/bmjopen-2019-032820 (PMC7712401; doi:10.1136/bmjopen-2019-032820)
Supplement: Author's manuscript [file bmjopen-2019-032820.draft_revisions.pdf]

BMJ Open

BMJ Open is committed to open peer review. As part of this commitment we make the peer review history of every article we publish publicly available.

When an article is published we post the peer reviewers' comments and the authors' responses online. We also post the versions of the paper that were used during peer review. These are the versions that the peer review comments apply to.

The versions of the paper that follow are the versions that were submitted during the peer review process. They are not the versions of record or the final published versions. They should not be cited or distributed as the published version of this manuscript.

BMJ Open is an open access journal and the full, final, typeset and author-corrected version of record of the manuscript is available on our site with no access controls, subscription charges or pay-per-view fees (http://bmjopen.bmj.com).

If you have any questions on BMJ Open's open peer review process please email info.bmjopen@bmj.com

BMJ Open

Making ICTs work for health: protocol for a mixed-methods study exploring processes for institutionalizing geo-referenced health information systems to strengthen MNCH service planning, referral and oversight in urban Bangladesh

Journal:	*BMJ Open*
Manuscript ID	bmjopen-2019-032820
Article Type:	Protocol
Date Submitted by the Author:	08-Jul-2019

Complete List of Authors:	Islam, Rubana ; University of New South Wales, School of Public Health and Community Medicine<br>Adams, Alayne; Georgetown University , Department of International Health ; BRAC University James P Grant School of Public Health<br>Hasan, Shaikh Mehdi ; International Centre for Diarrhoeal Disease Research Bangladesh<br>AHMED, RUSHDIA; International Centre for Diarrhoeal Disease Research Bangladesh<br>Bhattacharyya, Dipika; International Centre for Diarrhoeal Disease Research, Health Systems and Population Studies Division (HSPSD)<br>Shafique, Sohana; International Centre for Diarrhoeal Disease Research, Health Systems and Population Studies Division (HSPSD)

Keywords:	Urban health, maternal, neonatal and child health, information communication and technology, geographic information systems, health management information systems, Bangladesh

SCHOLARONE™ Manuscripts

I, the Submitting Author has the right to grant and does grant on behalf of all authors of the Work (as defined in the below author licence), an exclusive licence and/or a non-exclusive licence for contributions from authors who are: i) UK Crown employees; ii) where BMJ has agreed a CC-BY licence shall apply, and/or iii) in accordance with the terms applicable for US Federal Government officers or employees acting as part of their official duties; on a worldwide, perpetual, irrevocable, royalty-free basis to BMJ Publishing Group Ltd ("BMJ") its licensees and where the relevant Journal is co-owned by BMJ to the co-owners of the Journal, to publish the Work in this journal and any other BMJ products and to exploit all rights, as set out in our licence.

The Submitting Author accepts and understands that any supply made under these terms is made by BMJ to the Submitting Author unless you are acting as an employee on behalf of your employer or a postgraduate student of an affiliated institution which is paying any applicable article publishing charge ("APC") for Open Access articles. Where the Submitting Author wishes to make the Work available on an Open Access basis (and intends to pay the relevant APC), the terms of reuse of such Open Access shall be governed by a Creative Commons licence – details of these licences and which Creative Commons licence will apply to this Work are set out in our licence referred to above.

Other than as permitted in any relevant BMJ Author's Self Archiving Policies, I confirm this Work has not been accepted for publication elsewhere, is not being considered for publication elsewhere and does not duplicate material already published. I confirm all authors consent to publication of this Work and authorise the granting of this licence.

For peer review only

Title: Making ICTs work for health: protocol for a mixed-methods study exploring processes for institutionalizing geo-referenced health information systems to strengthen MNCH service planning, referral and oversight in urban Bangladesh

Authors

Rubana Islam (rubana.islam@student.unsw.edu.au)[1*]

Alayne Adams (alayne.adams@gmail.com)[2*]

Shaikh Mehdi Hasan (smehdi@icddrb.org)[3]

Rushdia Ahmed (ahmed.rushdia@yahoo.com)[3]

Dipika Shankar Bhattacharyya (dipikashankar@gmail.com)[3]

Sohana Shafique (sohana.shafique@icddrb.org)[3**]

Author Affiliations

[1] School of Public Health and Community Medicine, University of New South Wales, Sydney, Australia

[2] Department of Global Health, Georgetown University, Washington DC, USA

[3] Universal health Coverage Programme, Health Systems and Population Studies Division, icddr,b, Dhaka, Bangladesh

*These authors contributed equally for this work.

For peer review only - http://bmjopen.bmj.com/site/about/guidelines.xhtml

**Corresponding author/request for reprints:

Dr. Sohana Shafique

Assistant Scientist and Deputy Project Coordinator

Universal Health Coverage programme

Health Systems and Population Studies Division

icddr,b, Mohakhali, Dhaka. Bangladesh.

Email: sohana.shafique@icddrb.org

Phone: +880-2-9827001-10, ext. 2273

Word Count: 3961

Author Contributions

RI & AA conceptualized the study. RI & AA prepared the first draft of the manuscript. SMH, RA, DSB and SS revised the manuscript. RI & AA reviewed critically for important intellectual content; SS revised the version submitted with inputs from all other co-authors.

Acknowledgements

This research study was funded by International Development Research Centre (IDRC). icddr,b acknowledges with gratitude the commitment of International Development Research Centre (IDRC) to its research efforts. icddr,b is also grateful to the Governments of Bangladesh, Canada, Sweden and the UK for providing core/unrestricted support.

For peer review only - http://bmjopen.bmj.com/site/about/guidelines.xhtml

Competing interests

The authors declare no competing interests.

Funding

This work was supported by International Development Research Centre (IDRC), Canada (Grant Number: 108218-001).

Study period

2016-2019

Data statement section

Data will be handled according to the principles of the icddr,b policies and guidelines of the International Development Research Centre, Canada.

For peer review only

Abbreviations

ADB	Asian Development Bank
CCs	City Corporations
DGHS	Directorate General of Health Services
ERC	Ethical Review Committee
GIS	Geographic Information System
HMIS	Health Management Information System
ICT	Information Communication and Technology
IDI	In-depth Interviews
KII	Key Informant Interviews
LMICs	Low and Middle Income Countries
MNCH	Maternal, newborn and child health
MOHFW	Ministry of Health and Family Welfare
MOLGRDC	Ministry of Local Government, Rural Development and Cooperatives
NGOs	Non-Government Organizations
PHC	Primary Health Care
RRC	Research Review Committee
SDGs	Sustainable Development Goals
UHA	Urban Health Atlas
UHC	Universal Health Coverage
WHO	World Health Organization

For peer review only - http://bmjopen.bmj.com/site/about/guidelines.xhtml

Article Summary

Strengths and limitations of this study

- This mixed method implementation research is among the first in Bangladesh to explore processes for institutionalizing geo-referenced health information systems to strengthen MNCH service planning, referral and oversight in urban areas
- The proposed research will generate knowledge to enhance understanding of how geo-referenced health facility information can inform MNCH service planning and decision-making.
- This study is expected to provide valuable guidance beyond Bangladesh's HMIS on how to generate user buy-in, and policy uptake necessary to introduce MNCH-related ICTs in developing country contexts. The result of the study also might guide future intervention to scale up and sustain ICT tool into national HMIS
- This is a mixed-method implementation research applying pre-post design. Since it is not a quasi-experimental or Cluster randomized study, the evidence generated about the effectiveness of the intervention or proving causal relationship is difficult. Besides, the study is designed based on the consideration of local context of two municipalities outside Dhaka and two city corporations within Dhaka. There has been growing literature that the urban spectrum in Bangladesh is diverse in terms of population size, informal economy, public transport, and health outcomes. Therefore the result of this study might not be generalizable for all cities, but it will contribute to understand the urban context of Bangladesh.

For peer review only - http://bmjopen.bmj.com/site/about/guidelines.xhtml

- The sites selected for the study implementation is based on the availability of MFL and the UHA. Other cities, not included in the study, may have different experiences than the ones studied.
- Also, countries with different geo-political administration system, such as federal and state level divisions in governance, may face additional barriers in the institutionalization of the health ICTs. Hence the findings may not be widely generalizable. However, the insights generated will be relevant for health ICT implementation and can be built upon with further research both nationally and internationally.

For peer review only - http://bmjopen.bmj.com/site/about/guidelines.xhtml

ABSTRACT

Introduction: Disparities in health outcomes and access to maternal neonatal and child health (MNCH) are apparent among urban poor compared to national, rural or urban averages. A fundamental first step in addressing inequities in MNCH services is, knowing what services exist in urban areas, where these are located, who provides them, and who uses them. This study aims to institutionalize the Urban Health Atlas (UHA) - a novel ICT tool - to strengthen health service delivery and oversight and generate critical evidence to inform health policy and planning in urban Bangladesh.

Methods and analysis: This mixed-method implementation research will be conducted in purposively selected two City Corporations and two municipalities. Research activities will include an assessment of information needs and task review analysis of information users, stakeholder mapping, and cost estimation. To document stakeholder perceptions and experiences, Key Informant Interviews (KIIs) and In-depth Interviews (IDIs) will be conducted along with desk reviews to understand MNCH planning and referral decisions. The UHA will be refined to increase responsiveness to user needs and capacities and hands-on trainings will be provided to health managers. Cost estimation will be conducted to assess the financial implications regarding the uptake and scale up of UHA. Systematic documentation of the implementation process will be done. Policy decision-making and ICT health policy process flowcharts will be prepared using desk reviews and qualitative interviews. Thematic analysis using codes guided by WHO PATH toolkit and Policy Engagement Framework will be conducted for qualitative data. Stakeholder analysis will apply standard techniques and measurement scales. Descriptive analysis of quantitative data and cost estimation analysis will also be performed.

For peer review only - http://bmjopen.bmj.com/site/about/guidelines.xhtml

Ethics and dissemination: The study has been approved by the Institutional Review Board of icddr,b (# PR–16057). Study findings will be disseminated through national and international workshops, conferences, policy briefs and peer-reviewed publications.

Keywords

Urban health, maternal, neonatal and child health, information communication and technology, geographic information systems, health management information systems, Bangladesh.

For peer review only

INTRODUCTION

Bangladesh has embraced the Sustainable Development Goal-3 (SDGs) of achieving universal health coverage by 2030[1], however, there remain several challenges such as burgeoning population at all ages, pluralistic health systems, lack of governance and many more[2]. Despite the substantial progress Bangladesh has made in reducing maternal and child mortality[3], there are significant disparities in health-related outcomes and access to maternal neonatal and child health (MNCH) services along with socioeconomic and geographic dimensions. Health indicators are far worse in urban slums than the non-slum urban and the national average[4]. Nationally, mortality rate for children under five years of age is 65 per 1,000 live births while the rate is 81 per 1000 live births among urban slum residents in Bangladesh[4, 5]. Undergoing a rapid urbanization, Bangladesh projects by 2040 more than half of its citizens will reside in urban areas and almost one-third of them in slums[6]. Persistent inequities in key MNCH indicators in urban areas highlight the need to focus on issues of service coverage, access, quality and timely and appropriate referral as urgent policy priorities.

The Bangladesh urban health system is a smorgasbord of service providers with very little coordination and regulation[7, 8]. Several reasons have been proposed for inefficiencies in the system including poor planning and management capacity, poor coordination among the authorities, lack of clear, separate roles and responsibilities for the various authorities, service coverage gaps, and human resource management issues[9,10]. One consequence of the limited formal system of Primary Care Services in urban areas[11] is the emergence of the private sector in health including the proliferation of informal providers such as pharmacies. Currently, the formal private sector accounts for 80% of over 3500 hospitals in Bangladesh and lack of regulation of this sector has resulted in concerns about quality of care and accessibility,

For peer review only

especially for the urban poor, and presents challenges to moving towards universal health coverage[10].

A fundamental first step in addressing all these challenges in MNCH services in urban Bangladesh is an in-depth understanding of what services exist, their location, who provides them, and who utilizes them. A strong health management information system (HMIS), an essential component of sound programme development and implementation, can leverage such particulars as a requirement for strategic decision-making, better governance, and the basis upon which improved health outcomes depend[12, 13]. A Master Facility List (MFL) is a crucial constituent of HIS and permit the linkage of sub-systems in a national HIS architecture[14, 15]. Such MFL creation is being advocated by The World Health Organization (WHO) for ensuring better governance including systematic reporting and monitoring supervision[16,17].

Realizing the critical role that health information systems play in health management and building on political commitment towards “Digital Bangladesh”, the Bangladesh Directorate General of Health Services (DGHS), Ministry of Health and Family Welfare (MOHFW) is implementing the district health information software-2 (dhis2) with support from development partners. While the system has been rolled out nationally, information is largely confined to public health facilities in the country. With the exception of large public hospitals, and a handful of NGOs, data are currently unavailable for urban facilities in the system.

Addressing this gap, icddr,b has created geo-referenced heath facility databases for several major cities across Bangladesh. This dataset highlighted areas of service duplication and gaps in provision of MNCH services in poor urban settlements[18], enabled through the development of an Information Communication and Technology (ICT) tool called *Urban Health Atlas* (UHA)[19]. The UHA displays health facility data visually and permits their manipulation for

For peer review only - http://bmjopen.bmj.com/site/about/guidelines.xhtml

better planning and referral for urban health. One of the strengths of this dataset is inclusion of private sector in healthcare, from pharmacies to hospitals. The promise of UHA has intrigued multiple stakeholder groups, ranging from the Directorate General of Health Services (DGHS), local government, non-government organizations (NGOs), implementers and development partners. In the context of significant investments in urban health systems strengthening that are in pipeline, and absence of urban data in the national Management Information System (MIS), UHA is perceived timely and appropriate to be used and scaled up for guiding urban health planning process through institutionalizing such geo-referenced health facility information system. However, due to complexity of these kind of data, they risk being underutilized for health policy and planning unless specific efforts are attempted for making them more accessible to non-technical, policy and other local level stakeholders[20]. The purpose of this study, therefore, is to pilot the UHA for use in MNCH service delivery planning and referral, and by generating evidence on its utility, inform and strengthen advocacy for and action around its institutionalization into the government system with a view that the study fills an important information gap.

Study Aims

Three specific aims are identified for this study in order to institutionalize the UHA for MNCH service delivery, planning, and referral into the government system:

1. To document stakeholder perceptions and experiences in adopting a tool that enables use of health facility information for strategic planning, day-to-day decision-making, control and oversight, and improved administrative efficiency of urban MNCH services.

For peer review only - http://bmjopen.bmj.com/site/about/guidelines.xhtml

2. To identify policy and programmatic entry points that will facilitate broader use of geo-referenced health facility information and its regular update.
3. To estimate costs associated with bringing geo-referenced facility listing into the government system

METHODS

Study design and participants

The proposed implementation research employs a mixed method research approach to understand the processes by which an ICT tool that enables the visualization of health facility information, can contribute towards improving MNCH services for the urban poor. It will assess the uptake of UHA by the MNCH planners and position of the stakeholders and decision makers over a 3-year period from 2016-2019.

Theoretical frameworks

Two frameworks have been used to inform and guide this study: (i) WHO PATH toolkit[17]; and (ii) policy engagement framework[20]. The introduction of a new ICT tool is commonly accompanied by challenges that must be overcome. Before scaling up, it is important to conduct rigorous product planning and feasibility testing, and to identify and engage key stakeholders. In this regard, a toolkit has been published by WHO and PATH to guide the introduction and implementation of information and communications technology (ICT) in health information systems[17]. Drawing on this, three main phases of an ICT project is identified: pilot, scale, and sustain. In the pilot phase, the phase addressed in this study, a solution (UHA) is developed based on program needs and priorities, and tested on a small-scale to measure outcomes,

For peer review only - http://bmjopen.bmj.com/site/about/guidelines.xhtml

impacts, and costs, and identify potential improvements. Several other factors influence the introduction of the HMIS in developing countries including planning, stakeholders roles and responsibilities, cultural aspects, human capacity, financial aspects sustainability etc.[21], informed the first conceptual framework (figure 1).

In order to explore user perceptions, and policy and programmatic entry points, the policy engagement framework[20] will be employed to prospectively analyze policy that incorporates strategies for change. This framework will confer a systematic approach to the ongoing collection, analysis and use of political information (e.g. concerning actors, their interests, institutions, ideas, and policy processes and context) that can alter the balance of power between those in support of and those resisting change by enabling pro-reformers to intervene more effectively in the policy process[20].

Study sites

Two city corporations (CCs) – namely Dhaka North City Corporation (DNCC) and Dhaka South City Corporation (DSCC) and two municipalities Jessore and Dinajpur which have geo-referenced health facility data available from previous mapping exercise of icddr,b,[19] will be purposively selected. Jessore and Dinajpur municipalities will also be selected as they present marked differences from CCs in terms of size, structure, capacity and challenges posed.

Sample size

Through the stakeholder mapping, key urban MNCH decision-making actors at both national and local levels will be identified which will help identify potential users for UHA. They will be our

For peer review only - http://bmjopen.bmj.com/site/about/guidelines.xhtml

study respondents and training participants. The sampling strategy, type and number of respondents for each study activity are provided in table 1.

Table 1 Sampling strategy and sample type for each activity of the study

Activity and focus	Data collection methods	Sampling strategy	Respondent group	Sample size*
**Task Review** (document how MNCH planning and referral decisions are currently made)	KIIs	Opportunistic/ emergent sampling; Snowball sampling	–Urban health systems actors –National and local government officials –NGO programme managers	16
	Desk review	N/A	N/A	N/A
**User Need and User Experience** (explore user preferences, task needs and	IDIs (on user needs)	Opportunistic/ emergent sampling; Snowball sampling	–Policy makers within the MOHFW –Members of the Urban Health	16

For peer review only

experiences)			Cell of MOLGRDC<br>–Managers at City Corporations<br>–NGO programme managers	
	IDIs (on user experience)	Opportunistic/ emergent sampling; Snowball sampling	–Policy makers within the MOHFW<br>–Members of the Urban Health Cell of MOLGRDC<br>–Managers at City Corporations<br>–NGO programme managers	16
**Policy Engagement** (understand	KIIs	Opportunistic/ emergent	–Urban health systems actors	15

For peer review only - http://bmjopen.bmj.com/site/about/guidelines.xhtml

interactions between content, context, actors and processes of policy advocacy and entry points for sustainable incorporation of ICT into health systems)		sampling; Snowball sampling	–National and local government officials<br>–NGO programme managers	
	Desk review (urban health and ICT related policies)	N/A	N/A	N/A

ICT, Information Communication and Technology; IDI, In-depth Interviews; KII, Key Informant Interviews; MNCH, Maternal, newborn and child health; MOHFW, Ministry of Health and Family Welfare; MOLGRDC, Ministry of Local Government, Rural Development and Cooperatives.

Implementation procedure of the pilot

The study implementation is envisaged with stakeholder sensitization and developing partnership with government. This is critical because the research will closely work with government health system in urban areas, so for its successful implementation. In addition to this, there will be intervention to conduct capacity building sessions for the government managers and actors of urban health around the use of the UHA. The details of these activities are provided below.

For peer review only - http://bmjopen.bmj.com/site/about/guidelines.xhtml

Stakeholder consultation and engagement: Two stakeholder consultation workshops will be carried out to identify and engage key stakeholders for advocacy to avoid failure and resource wastage. Detail information on identification and mapping of stakeholders has been described in the data collection section. Ideas will be generated for uptake, regular use, and update of UHA.

Partnership development with Government: For implementation partnership with Management Information System (MIS), DGHS, MOHFW will be developed and a Memorandum of Understanding will be signed between icddr,b and DGHS. Permission letters will also be obtained from mayors of CCs and municipalities.

Capacity building on UHA: Hands-on UHA training workshops will be organized for stakeholders involved in urban MNCH services planning and implementation. During and following these workshops, participants will conduct several applied exercises to further consolidate skills and comfort in using UHA. A total of 6 trainings will be conducted in three field sites and for each training session 20-22 participants will be invited. The participants will be from policy makers within the Ministry of Health and Family Welfare (MOHFW), members of the Urban Health Cell of Ministry of Local Government, Rural Development and Cooperatives (MOLGRD&C), Chief Health Officers at City Corporations, NGO programme managers.

For peer review only - http://bmjopen.bmj.com/site/about/guidelines.xhtml

Modification ICT tool: Based on the feedback received from stakeholders as well as the study participants the UHA will be modified throughout the study. A prototype of a mobile application will be developed and feasibility of this application will be explored.

Data collection

Interviews will be conducted by an experienced group of researchers trained in qualitative interviewing. The team consists of a mixed method expert, a qualitative & ethnography expert and two software programmers, one GIS expert and an economist. The team will begin data collection in Dhaka (both city corporations), then move to the municipalities to ensure the convenience in terms of time and travelling. A period of rapport building with key stakeholders in each study site will be critical to the success of this research given known difficulties in accessing the Government sector with their workload. Networks and negotiation will be important in opening doors and initiating discussion. The UHA tool will be assessed for impact on MNCH decision-making and outcomes. It is unlikely, however, that these effects would be apparent within three years of implementation. Thus, success of the tool will be determined based on user experiences as specified by WHO PATH Toolkit i.e. better indicators for strategic planning, day-to-day decision-making, control and oversight, and reduced administrative burden.

Data collection methods for objective 1

To address objective 1, assessment of information needs and task review analysis will be done by desk review, in-depth interviews and click streams. Guidelines for **qualitative interviews** will be developed based on the WHO PATH toolkit's questions to measure success. Three qualitative research activities are envisaged to address this objective:

For peer review only - http://bmjopen.bmj.com/site/about/guidelines.xhtml

1. Key informant interviews (KIIs) with urban health system actors along with desk reviews to understand and document how MNCH planning and referral decisions are currently made.
2. In-depth interviews (IDIs) with potential UHA users to explore user preferences and task needs to refine the tool in advance of training.
3. IDIs with UHA users to understand their experiences and to document challenges and successes of using UHA for MNCH service decision-making during training and one- & three-months post-training.

In addition to qualitative assessments of user experiences, how different stakeholders are using data remotely through quantitative assessments of (i) User's click streams; (ii) Task time devoted to different applications will be monitored. Written feedback through online tools (i.e. Google Analytics) that facilitate remote testing will also be collected and further used to generate more user-friendly functions that meet user needs.

Data collection methods for objective 2

To identify entry points that will facilitate broader use of geo-referenced facility information and its regular update, stakeholder mapping, policy mapping using desk review, KIIs, and stakeholder consultation workshop will be conducted. Guidelines for qualitative interviews will be developed using the policy engagement framework as a guide. Policy and programmatic entry points for the broader use and update of facility information, stakeholder analysis will be undertaken using Policy Engagement Framework as a guide. Stakeholders are identified as persons, groups, organization members or systems that affect or can be affected by a

For peer review only - http://bmjopen.bmj.com/site/about/guidelines.xhtml

project/program/activity. Accordingly, stakeholder analysis is an approach for generating knowledge about roles, behavior, inter-relation and intention of associated actors and their influence in implementation processes of a program or policy[22]. Given the importance of stakeholder satisfaction and support for the success of any program,[23,24] incorporating stakeholders' perspectives and needs is a critical step in gaining ownership around an ICT innovation like UHA and its incorporation into routine information systems, and use for decision-making. Following qualitative methods will be used to fulfil objective 2:

- Stakeholder mapping including the identification and listing of stakeholder groups involved in urban health based on available literature and expert opinion.
- A semi-structured guideline will be used to collect information during stakeholder consultation workshops to explore their respective interests, roles and responsibilities in urban health, their information needs, and perceptions of how they can contribute to institutionalizing UHA.
- KIIs along with desk reviews to understand the processes of current health policy-making mechanisms and what other policies affect the integration of ICT in health.

Data collection methods for objective 3

To estimate cost of bringing facility listing into the government information system total cost of ownership for UHA development and implementation will be estimated using ingredient approach. Data will be collected through structured questionnaire, document review and KII. Cost will be estimated only supply side aspects. The budget matrix will be developed with cost drivers proposed in the WHO PATH toolkit.

For peer review only - http://bmjopen.bmj.com/site/about/guidelines.xhtml

The cost for development and implementation of Urban Health Atlas (UHA) tools, coordination, and engagement of city corporations and DGHS will be estimated. Both financial costs and economic costs of the program shall be estimated. Financial costs represent the actual expenditures on goods and services purchased. Economic costs include the estimated value of goods or services for which either there are no financial transactions or the price of a specific good did not reflect the cost of using it productively elsewhere[25]. The cost will be separated for start-up cost and implementation cost. The implementation cost comprise the costs required to run and for regular maintenance of the ICT tools while executing intervention[26].

All supply-side inputs will be identified, quantified and valued through facility-level inventory, record review and key informant interview. Fixed cost and variable cost will be captured. Shared cost items, including salary, buildings, furniture, supervision, transportation, and vehicles, will be identified through observation and interviews of relevant personnel. The shared costs will be apportioned by proportion of the time-involvement of the relevant items (like, office rent, common vehicle) to different activities. The time of the volunteers will be transformed into costs by using the minimum wage level of manual workers in Bangladesh. There are some examples of components included in ingredient approaches (table 2)[26, 27]:

Table 2 Components included in ingredient approaches for cost

Method Name	Methodology	Inputs	Activities
Ingredients approach	Quantities x price, personnel, percentage use	personnel, ICT tools, vehicles,	Personnel , ICT tools, vehicles

For peer review only - http://bmjopen.bmj.com/site/about/guidelines.xhtml

ICT, Information Communication and Technology.

To identify activities of the UHA tool implementation and their related inputs, a review of program documents and interviews with relevant personnel will be conducted. The unit price/salary information will be collected from responsible program management. In case of missing unit price of any items, the market price of those items will be collected. Semi-structured checklists for cost data collection will be developed considering the program context and using WHO PATH toolkit as a guiding framework. The budget matrix will be completed with help from key personnel associated with costing and budgeting identified during the stakeholder mapping and research team's own estimates.

Data analysis

A process flowchart for current decision-making practices will be prepared using the KII and Organizational process reviews. A list of user needs will be made and shared at a stakeholder consultation meeting to identify the most important and feasible functions to be added to UHA.

For *qualitative data*, an outline plan for data analysis will be prepared in advance of research along with a priori codes. These codes, mostly focusing on user experience, will be derived from the WHO PATH toolkit[17]. The analysis will be open to emerging themes as well. All interviews will be recorded provided consent has been obtained, but with simultaneous note taking in case of equipment failure. Data transcription will occur immediately following each interview, followed by translation. Data familiarization will involve reading transcripts repeatedly to surface emerging themes and identify any missed opportunities for further

For peer review only - http://bmjopen.bmj.com/site/about/guidelines.xhtml

exploration. Transcripts will be coded using Atlas-ti (version-7.5.7). A team approach to analysis will be employed to minimize individual biases. Inter-coder reliability will be checked. Group discussions of emerging themes and patterns in the data will be tested using data displays that allow more systematic pattern-testing across respondents.

For *stakeholder analysis*, stakeholders' influence, importance and agreement will be explored applying standard techniques and measurement scales mentioned in table 3**.** A position diagram with level of agreement and level of influence will also be plotted to identify stakeholders who are already convinced to work and help institutionalize UHA and who need to be brought into agreement.

For peer review only

Table 3 Operational definitions for stakeholder analysis for policy engagement

Theme	Terms used	Operational definition
**Influence, importance and agreement analysis of stakeholders**	Level of influence	Stakeholders' influence will be determined according to each stakeholder group's perception and views on who is important in terms of urban health care delivery.
	Level of agreement	Stakeholders' agreement will be determined according to how much each stakeholder agreed.
	Level of importance	The stakeholders' importance will be determined according to how important each stakeholder group is to the other groups.
**Power and**	Overall power	Power of a stakeholder-group will be assessed as

For peer review only - http://bmjopen.bmj.com/site/about/guidelines.xhtml

**leadership**		compared to all other groups in Bangladeshi urban healthcare delivery system. Power of stakeholders will be measured as the product of multiplication of influence and importance.
	Relative position	Relative position of each stakeholder group will be assessed by comparing one group's position to other groups in broader scenario.
**Relative positions of stakeholders**	Drivers	Stakeholders who have high level of importance as well as high level of influence on public sector health care delivery system
	Supporters	Stakeholders who have high level of importance but low level of influence on urban healthcare delivery system
	Bystanders	Stakeholders who have low level of importance and low level of influence on urban healthcare delivery system
	Abstainers	Stakeholders who have no influence and no importance on urban healthcare delivery system
	Blockers	Stakeholders who have low level of importance but high level of influence on urban healthcare care delivery system.

For peer review only

For *policy engagement analysis,* results from stakeholder analysis will be used to identify links between actors in the process of implementation and uptake. KIIs conducted will contribute towards a comprehensive understanding of interactions between content, context, actors and processes of policy advocacy and entry points crucial for sustainable incorporation of ICT in health systems. Interviews will be analyzed using a priori codes drawn from the Policy Engagement Framework. Emphasis will be given on the processes of current health policy development mechanisms and effect of other policies for integration of ICT in health. A Health Policy for ICT process flowchart will be prepared. Information on how context is considered and dealt with when policies are formed and what processes need to be changed to integrate UHA into the system according to policy engagement framework[20].

For *quantitative data*, simple descriptive analysis will be performed to show user rates by different stakeholders. A set of parameters to be analyzed are: number of users who accessed UHA, type of users, scope of UHA use, and types of problems faced. For data analysis, software like MS Excel and STATA will be used as appropriate.

Cost of implementing UHA will be estimated using a direct approach. Average cost for each activity will be calculated. All supply-side inputs will be identified, quantified and valued through record review and KIIs. Shared cost items, including salary, buildings, furniture, supervision, transportation, and vehicles will be identified through observation and interviews of relevant personnel. By considering the nature of inputs, these will be categorized into capital, recurrent as well as fixed and variable cost items. Shared costs will be apportioned by proportion of the time-involvement of the relevant items (office rent, common vehicle) to

For peer review only - http://bmjopen.bmj.com/site/about/guidelines.xhtml

different activities. The inputs will be identified using discussion with relevant personnel, observation and record review. If unavailable, market prices will be applied to estimate costs. The capital items will be annualized, and common costs will be apportioned as per requirement. Annual values of capital items will be estimated from their expected useful life years and annuitization will be done using 3% discounting rate whenever applicable[28]. The study will allocate the cost for shared items (e.g. office space, appliances) by using actual utilization of items for this activity. Utilization information of the shared items will be collected from responsible project staff.

Finally, the cost for shared items will be estimated by multiplying percentage-use information with the total cost of the items. Total cost will be calculated by summing up the start-up and implementation costs. Relative contribution of start-up and implementation cost will be calculated. The cost-drivers in each activity will be identified considering the larger share of total cost.

Process documentation

In addition to all these activities, the study investigators will systematically document the implementation process for policy uptake and institutionalization of UHA, focusing particularly on the contextual factors and their influence on implementation process using a process documentation template. It will be a continuous information gathering process during the project period. Information will be gathered on different approaches including – field-level activities, meetings, negotiations, decision-taken, planning, implementation of decisions, resolution of differences etc.

For peer review only - http://bmjopen.bmj.com/site/about/guidelines.xhtml

Among various methods for process documentation, this study will employ three:

- Documents such as meeting notes, list of Technical Advisory Group (TAG) members, TAG lists, Terms of References (ToR) for TAG, field-diaries of project staff outlining their observations
- Images of stakeholder consolation workshop, training and other project activities.
- Recordings of interviews, trainings, meetings, workshops etc.

This process documentation of the pilot phase for UHA institutionalization will generate supporting knowledge to be applied for the next two phases identified by the WHO/PATH toolkit – scale up and sustain.

Patient and public involvement

There will be no direct patient or public involvement in this implementation research. However, a Technical Advisory Group (TAG) will be formulated for project governance, which will consist of representatives from Government, development partners, NGOs, academicians and senior researchers and urban health actors. Regular meetings will be held with partners and staff for problem solving. At the end of the study, the TAG will comment on the study findings and contribute to the dissemination plan.

Ethics

This study has received ethical approval from the Research Review Committee (RRC) and the Ethical Review Committee (ERC) of icddr,b for critical review of technical competencies in-

For peer review only - http://bmjopen.bmj.com/site/about/guidelines.xhtml

depth examination of ethical issues related to local context respectively. As the study involves key stakeholders within urban health systems, poses no more than minimal risk to subjects. Participants will be asked for written consent prior interviewing and will remain anonymous and unidentifiable. Tape recorders will be used for recording the discussions in order to collect full and intact thoughts after obtaining consent. All other form of data will be kept in locked storage, or controlled access folders, allowing only investigators of the study and members of the ERC of icddr,b to access information, if needed.

Dissemination

Findings from this research will be disseminated at various levels to develop interest and support from a wide variety of audiences i.e. public, private, NGO, civil society and donors. In doing so, we hope to build a diverse constituency of individuals and organizations willing and able to translate evidence yielded by the study, into policy action.

Local dissemination: Findings will be presented to relevant local administrators, development partners and NGOs and other relevant parties (local health practitioners), researchers.

National dissemination: A series of interactive workshops and briefing sessions with various stakeholders will be arranged to create linkage with national fora. The main aim will be to translate findings in a more visual and engaging format; i.e. research briefs and interactive project brochures, to reach a range of stakeholders.

For peer review only

International dissemination: This will include publishing findings in peer-reviewed journals and presenting in scientific forums, conferences and symposiums and linking with international learning platforms. The main objective is contributing to the global knowledge pool.

CONCLUSIONS

The proposed study seeks to understand the utility and processes by which the visualization of geo-referenced health facility information can contribute towards improving MNCH services in terms of strategy development, decision-making and oversight, with a special focus on the urban poor, and how best to approach the existing system for its institutionalization. The results from this study will inform efforts to scale up the generation and application of facility listing data in urban areas nationwide. Beyond Bangladesh, this study is also expected to provide valuable guidance on how to generate user buy-in, and policy uptake necessary to introduce, scale-up, and sustain MNCH-related ICTs in similar LMICs.

For peer review only

REFERENCES

1. Adams AM, Ahmed SM, Evans TG. Universal health care in Bangladesh–promises and perils. *Lancet Glob Health* 2018;6: e10-11.
2. Ahmed, Syed Masud, Faizul Islam, Kuhel, Bhuiya, Abbas eds. *Bangladesh Health Watch Report 2014 Urban Health Scenario: Looking Beyond 2015.* Dhaka, Bangladesh: James P Grant School of Public Health, BRAC University 2015.
3. Chowdhury AMR, Bhuiya A, Chowdhury ME, et al. The Bangladesh paradox: exceptional health achievement despite economic poverty. *Lancet* 2013;382: 1734-1745.
4. National Institute of Population Research and Training (NIPORT), International Centre for Diarrhoeal Disease Research, Bangladesh (icddr,b), MEASURE Evaluation. *Bangladesh Urban Health Survey 2013 Final Report*. Dhaka, Bangladesh and Chapel Hill, North Carolina, USA: NIPORT, icddr,b and Measure Evaluation 2013. p322.
5. National Institute of Population Research and Training (NIPORT), Mitra and Associates, and ICF International. *Bangladesh Demographic and Health Survey 2014: Key Indicators*. Dhaka, Bangladesh, and Rockville, Maryland, USA: NIPORT, Mitra and Associates, and ICF International 2016.
6. United Nations. Department of Economic and Social Affairs. World Urbanization Prospects: The 2018 Revision. [Key Facts]. 2018. https://esa.un.org/unpd/wup/publications/Files/WUP2018-KeyFacts.pdf
7. Alam SMN. Health service delivery: the state of government-non-government relations in Bangladesh. *Public Adm Dev* 2011;31:273-81.
8. Afsana K, Wahid SS. Health care for poor people in the urban slums of Bangladesh. *Lancet*. 2013;382:2049-51.

For peer review only - http://bmjopen.bmj.com/site/about/guidelines.xhtml

9. Huque R, Barkat A, Sabina N. *Public health expenditure: equity, efficacy and universal health coverage*. In: Ahmed SM, Mahmud S, Evans TG, et al. eds. *Bangladesh Health Watch Report 2011 Moving towards Universal Health Coverage*. Mohakhali, Bangladesh: James P Grant School of Public Health, BRAC University 2012:25.
10. Osman F. Public health, urban governance and the poor in Bangladesh: policy and practice. *Asia-Pacific Development Journal* 2009;16:27-58.
11. Local Government Division, Government of the People's Republic of Bangladesh. Urban Primary Health Care Services Delivery Project 2014. http://uphcp.gov.bd/.
12. Nutley T, Reynolds HW. Improving the Use of Health Data for Health System Strengthening. *Glob Health Action* 2013;6.
13. Few S. Data *Visualization for Human Perception*. In: The Encyclopedia of Human – Computer Interaction, 2nd Ed. Aarhus, Denmark: The Interaction Design Foundation 2014.
14. World Health Organization. *Creating a Master Health Facility List*. Geneva, Switzerland: World Health Organization 2013.
15. Rose-Wood A, Heard N, Thermidor R, et al. Development and use of a master health facility list: Haiti's experience during the 2010 earthquake response. *Glob Health Sci Pract* 2014;2:357-365.
16. World Health Organization. *Toolkit on monitoring health systems strengthening Measuring Health Systems Strengthening and Trends: A Toolkit for Countries*. World Health Organization 2008.
17. World Health Organization, PATH. *Planning an Information Systems Project: A Toolkit for Public Health Managers*. Seattle: PATH 2013.

For peer review only - http://bmjopen.bmj.com/site/about/guidelines.xhtml

18. Adams A.M., Ahmed S., Hasan S.M. et al. *Mapping the Urban Healthcare Landscape in 5 City Corporations, Bangladesh*. Mohakhali, Dhaka: icddr,b 2015.

19. icddrb. The Urban Health Atlas. http://www.urbanhealthfacilities.icddrb.org.

20. Buse K, Booth D, Murindwa G, et al. *Donors and the Political Dimensions of Health Sector Reform: The Cases of Tanzania and Uganda*. London, UK: Overseas Development Institute 2008.

21. Archangel N. *The critical issues affecting the introduction of Health Management Information Systems in developing countries in Africa*. Amsterdam: Universiteit van Amsterdam 2007.

22. Bryson JM. What to do when stakeholders matter: stakeholder identification and analysis techniques. *Public Management Review*. 2004;6:21-53.

23. Bryson JM. *Strategic planning for public and nonprofit organizations: A guide to strengthening and sustaining organizational achievement (Vol 1).* San Francisco, CA: Jossey-Bass Publishers 1995.

24. Moore MH. *Creating public value: Strategic management in government*. Cambridge, MA: Harvard University Press 1995.

25. Gold MR, Siegel JE, Russell LB, et al. eds. *Cost-effectiveness in Health and Medicine 1ed*. New York: Oxford University Press 1996.

26. Drummond MF, Sculpher MJ, Claxton K, et al. *Methods for the economic evaluation of health care programmes*. Oxford University Press 2015.

27. World Health Organization. *Comprehensive Multi-Year Planning (cMYP): A Tool and User Guide for cMYP Costing and Financing*. Geneva, Switzerland: World Health Organization 2014.

For peer review only - http://bmjopen.bmj.com/site/about/guidelines.xhtml

28. Fiedler JL, Villalobos CA, De Mattos AC. An activity-based cost analysis of the Honduras community-based, integrated child care (AIN-C) programme. *Health Policy Plan* 2008;23:408-427.

For peer review only

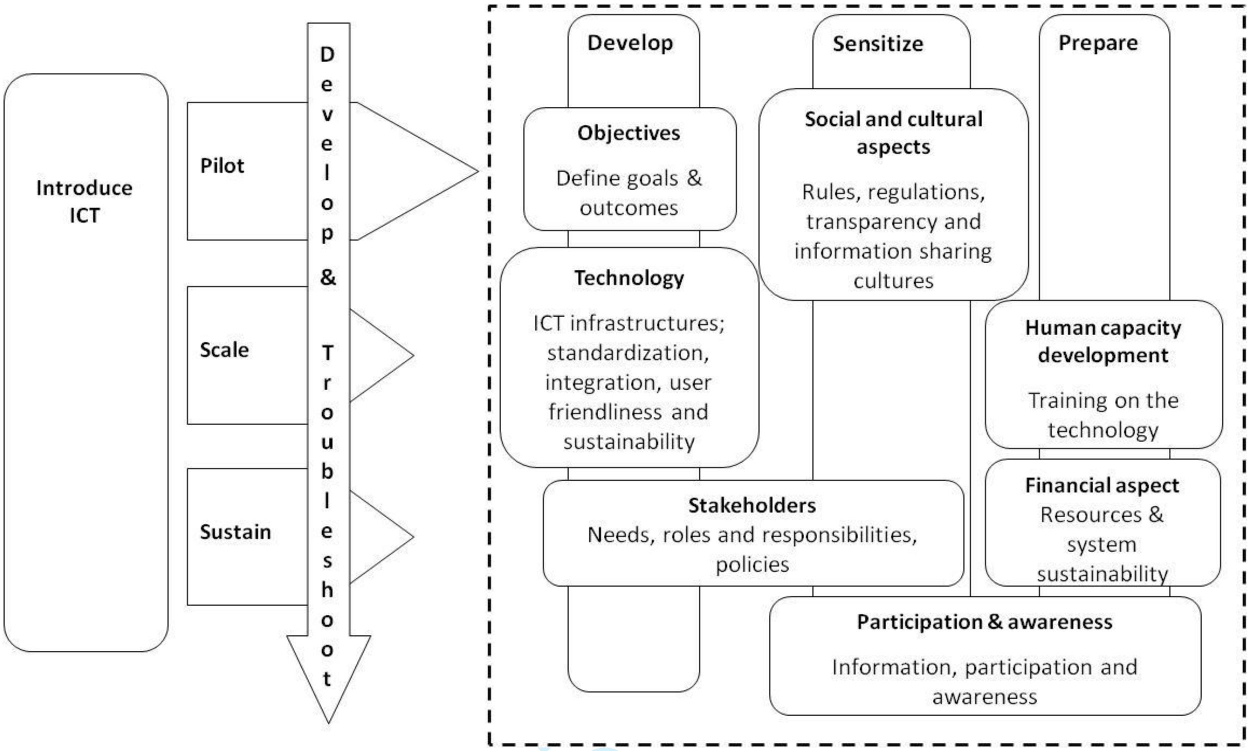

Figure 1. Modified Conceptual framework for Information Communication and Technology (ICT) implementation in developing countries

review only

BMJ Open

Making Information and Communications Technologies (ICTs) work for health: protocol for a mixed-methods study exploring processes for institutionalizing geo-referenced health information systems to strengthen Maternal Neonatal and Child Health (MNCH) service planning, referral and oversight in urban Bangladesh

Journal:	*BMJ Open*
Manuscript ID	bmjopen-2019-032820.R1
Article Type:	Protocol
Date Submitted by the Author:	11-Mar-2020
Complete List of Authors:	Islam, Rubana ; University of New South Wales, School of Public Health and Community Medicine<br>Adams, Alayne; McGill University, Department of Family Medicine; BRAC University James P Grant School of Public Health<br>Hasan, Shaikh Mehdi ; International Centre for Diarrhoeal Disease Research Bangladesh, Health Systems and Population Studies Division<br>AHMED, RUSHDIA; International Centre for Diarrhoeal Disease Research Bangladesh, Health Systems and Population Studies Division<br>Bhattacharyya, Dipika Shankar; International Centre for Diarrhoeal Disease Research, Health Systems and Population Studies Division<br>Shafique, Sohana; International Centre for Diarrhoeal Disease Research, Health Systems and Population Studies Division
**Primary Subject Heading**:	Public health
Secondary Subject Heading:	Global health, Health policy, Health informatics, Health services research
Keywords:	Urban health, maternal, neonatal and child health, information communication and technology, geographic information systems, health management information systems, Bangladesh

SCHOLARONE™
Manuscripts

I, the Submitting Author has the right to grant and does grant on behalf of all authors of the Work (as defined in the below author licence), an exclusive licence and/or a non-exclusive licence for contributions from authors who are: i) UK Crown employees; ii) where BMJ has agreed a CC-BY licence shall apply, and/or iii) in accordance with the terms applicable for US Federal Government officers or employees acting as part of their official duties; on a worldwide, perpetual, irrevocable, royalty-free basis to BMJ Publishing Group Ltd ("BMJ") its licensees and where the relevant Journal is co-owned by BMJ to the co-owners of the Journal, to publish the Work in this journal and any other BMJ products and to exploit all rights, as set out in our licence.

The Submitting Author accepts and understands that any supply made under these terms is made by BMJ to the Submitting Author unless you are acting as an employee on behalf of your employer or a postgraduate student of an affiliated institution which is paying any applicable article publishing charge ("APC") for Open Access articles. Where the Submitting Author wishes to make the Work available on an Open Access basis (and intends to pay the relevant APC), the terms of reuse of such Open Access shall be governed by a Creative Commons licence – details of these licences and which Creative Commons licence will apply to this Work are set out in our licence referred to above.

Other than as permitted in any relevant BMJ Author's Self Archiving Policies, I confirm this Work has not been accepted for publication elsewhere, is not being considered for publication elsewhere and does not duplicate material already published. I confirm all authors consent to publication of this Work and authorise the granting of this licence.

For peer review only

Title: Making Information and Communications Technologies (ICTs) work for health: protocol for a mixed-methods study exploring processes for institutionalizing geo-referenced health information systems to strengthen Maternal Neonatal and Child Health (MNCH) service planning, referral and oversight in urban Bangladesh

Authors

Rubana Islam (rubana.islam@student.unsw.edu.au)[1*]

Alayne Adams (alayne.adams@gmail.com)[2*]

Shaikh Mehdi Hasan (smehdi@icddrb.org)[3]

Rushdia Ahmed (ahmed.rushdia@yahoo.com)[3]

Dipika Shankar Bhattacharyya (dipikashankar@gmail.com)[3]

Sohana Shafique (sohana.shafique@icddrb.org)[3**]

Author Affiliations

[1] School of Public Health and Community Medicine, University of New South Wales, Sydney, Australia

[2] Department of Global Health, Georgetown University, Washington DC, USA

[3] Universal Health Coverage Programme, Health Systems and Population Studies Division, icddr,b, Dhaka, Bangladesh

*These authors contributed equally for this work.

For peer review only - http://bmjopen.bmj.com/site/about/guidelines.xhtml

**Corresponding author/request for reprints:

Dr. Sohana Shafique

Assistant Scientist and Deputy Project Coordinator

Universal Health Coverage programme

Health Systems and Population Studies Division

icddr,b, Mohakhali, Dhaka. Bangladesh.

Email: sohana.shafique@icddrb.org

Phone: +880-2-9827001-10, ext. 2273

Word Count: 4550

Author Contributions

RI & AA conceptualized the study. RI & AA prepared the first draft of the manuscript. SMH, RA, DSB and SS revised the manuscript. RI & AA reviewed critically for important intellectual content; SS revised the version submitted with inputs from all other co-authors.

Acknowledgements

This research study was funded by International Development Research Centre (IDRC). icddr,b acknowledges with gratitude the commitment of International Development Research Centre (IDRC) to its research efforts. icddr,b is also grateful to the Governments of Bangladesh, Canada, Sweden and the UK for providing core/unrestricted support.

For peer review only - http://bmjopen.bmj.com/site/about/guidelines.xhtml

Competing interests

The authors declare no competing interests.

Funding

This work was supported by International Development Research Centre (IDRC), Canada (Grant Number: 108218-001).

Study period

2016-2019

Data statement section

Data will be handled according to the principles of the icddr,b policies and guidelines of the International Development Research Centre, Canada.

For peer review only

Abbreviations

ADB	Asian Development Bank
CCs	City Corporations
DGHS	Directorate General of Health Services
ERC	Ethical Review Committee
GIS	Geographic Information System
HMIS	Health Management Information System
ICT	Information Communication and Technology
IDI	In-depth Interviews
KII	Key Informant Interviews
LMICs	Low and Middle Income Countries
MNCH	Maternal, newborn and child health
MOHFW	Ministry of Health and Family Welfare
MOLGRDC	Ministry of Local Government, Rural Development and Cooperatives
NGOs	Non-Government Organizations
PHC	Primary Health Care
RRC	Research Review Committee
SDGs	Sustainable Development Goals
UHA	Urban Health Atlas
UHC	Universal Health Coverage
WHO	World Health Organization

For peer review only

For peer review only - http://bmjopen.bmj.com/site/about/guidelines.xhtml

ABSTRACT

Introduction: Disparities in health outcomes and access to maternal neonatal and child health (MNCH) are apparent among urban poor compared to national, rural or urban averages. A fundamental first step in addressing inequities in MNCH services is, knowing what services exist in urban areas, where these are located, who provides them, and who uses them. This study aims to institutionalize the Urban Health Atlas (UHA) - a novel ICT tool - to strengthen health service delivery and oversight and generate critical evidence to inform health policy and planning in urban Bangladesh.

Methods and analysis: This mixed-method implementation research will be conducted in four purposively selected urban sites representing larger and smaller cities. Research activities will include an assessment of information needs and task review analysis of information users, stakeholder mapping, and cost estimation. To document stakeholder perceptions and experiences, Key Informant Interviews (KIIs) and In-depth Interviews (IDIs) will be conducted along with desk reviews to understand MNCH planning and referral decisions. The UHA will be refined to increase responsiveness to user needs and capacities, and hands-on training will be provided to health managers. Cost estimation will be conducted to assess the financial implications of UHA uptake and scale-up. Systematic documentation of the implementation process will be undertaken. Policy decision-making and ICT health policy process flowcharts will be prepared using desk reviews and qualitative interviews. Thematic analysis of qualitative data will involve both emergent and a priori coding guided by WHO PATH toolkit and Policy Engagement Framework. Stakeholder analysis will apply standard techniques and measurement scales. Descriptive analysis of quantitative data and cost estimation analysis will also be performed.

For peer review only - http://bmjopen.bmj.com/site/about/guidelines.xhtml

Ethics and dissemination: The study has been approved by the Institutional Review Board of icddr,b (# PR–16057). Study findings will be disseminated through national and international workshops, conferences, policy briefs and peer-reviewed publications.

Keywords

Urban health, maternal, neonatal and child health, information communication and technology, geographic information systems, health management information systems, Bangladesh.

Article Summary

Strengths and limitations of this study

- This mixed method implementation research is among the first in Bangladesh to explore processes for institutionalizing geo-referenced health information systems to strengthen MNCH service planning, referral and oversight in urban areas
- The proposed research will generate knowledge to enhance understanding of how geo-referenced health facility information can inform MNCH service planning and decision making.
- This study is expected to provide valuable guidance beyond Bangladesh's HMIS on how to generate user buy-in, and policy uptake necessary to introduce MNCH-related ICTs in developing country contexts.
- The impact of this information system tool on health outcomes cannot be established through this study.

For peer review only

INTRODUCTION

Bangladesh has embraced the Sustainable Development Goal-3 (SDGs) of achieving universal health coverage by 2030,[1] however, challenges related to rapid population growth, pluralistic health systems, and lack of governance, among others, are substantial.[2] Although Bangladesh has made extraordinary progress in reducing maternal and child mortality,[3] there are significant disparities in health-related outcomes and access to maternal neonatal and child health (MNCH) services stratified along both socioeconomic and geographic dimensions. Health indicators are far worse in urban slums than both non-slum urban areas and the national average.[4] Nationally, the mortality rate for children under five years of age is 65 per 1,000 live births and 49 per 1,000 live births in rural areas while the rate is 81 per 1000 live births among urban slum residents.[4, 5] Undergoing rapid urbanization, the country is projected to become over 50% urban by 2040, with almost one-third of urban residents living in slums.[6] Persistent inequities in key MNCH indicators in urban areas highlight the need to focus on issues of service coverage, access, quality and timely and appropriate referral as urgent policy priorities.

The Bangladesh urban health system is a smorgasbord of service providers characterized by inadequate coordination and regulation, and geographic and socioeconomic inequities in healthcare access.[7, 8] Several reasons have been proposed for inefficiencies in the system including poor planning and management capacity, weak coordination among the authorities, lack of clear, separate roles and responsibilities for the various authorities, service coverage gaps, and human resource management issues.[9, 10] Of particular concern in urban areas is the lack of adequate public primary care infrastructure and services which disproportionately impacts the urban poor, and poses significant challenges to the country's aspirations to meet the goal of Universal Health Coverage by 2030.[11] One consequence of limited formal primary healthcare

For peer review only - http://bmjopen.bmj.com/site/about/guidelines.xhtml

services in urban areas[12] is the emergence of the private sector in health including the proliferation of informal providers such as pharmacies on which many of the urban poor rely. The formal private sector is equally massive, accounting for 80% of over 3500 hospitals in Bangladesh, and an even greater percentage in urban areas. Lack of regulation of this sector has resulted in concerns about quality of care and financial accessibility, especially for the urban poor.[10]

A fundamental first step in addressing inequities in urban healthcare access is an in-depth understanding of what services exist, their location, who provides them, and who utilizes them. A strong health management information system (HMIS), an essential component of sound programme development and implementation. Enabling the use of data for strategic decision-making, better governance, institutionalized HMIS systems represent the foundation upon which improvements in health outcomes can be monitored and greater accountability ensured.[13, 14] A Master Facility List (MFL) is a crucial constituent of HIS and permits the linkage of sub-systems within national HIS architecture.[15,16] MFL is advocated by The World Health Organization (WHO) as an effective means of ensuring better governance including systematic reporting and monitoring supervision.[17,18] MFLs like UHA are expected to facilitate health service planning and management through mapping or visualizing the distribution of health services and resources. It can also assist health service providers in identifying appropriate referral facilities for patients.[19] These functions can help improve equitable service coverage and reduce delays in receiving appropriate care, which in turn can impact health outcomes such as maternal and child mortality among the urban poor. A theory of change is provided in supplemental file 1.

For peer review only

Realizing the critical role that health information systems play in health management and building on political commitment towards "Digital Bangladesh", the Bangladesh Directorate General of Health Services (DGHS), Ministry of Health and Family Welfare (MOHFW) is implementing the District Health Information Software-2 (DHIS2) with support from development partners. While the system has been rolled out nationally, information is largely confined to public healthcare facilities. In urban areas, with the exception of large public hospitals and a number of NGOs involved in primary care provision, data are particularly sparse, especially for the massive private sector.

Urban Health Atlas: A novel ICT tool

Addressing this information gap, icddr,b has created a geo-referenced heath facility database for nine major cities and municipalities across Bangladesh. This dataset consists of a census of all healthcare facilities and the services they provide along with their geo locations.[20] To enable the practical application of this dataset, an Information Communication and Technology (ICT) tool called *Urban Health Atlas* (UHA) was developed (http://urbanhealthatlas.com).[21] This GIS-based interactive online tool displays health facility data visually and permits their manipulation for better healthcare planning and decision making. Providing detailed information on the location and services available at public and private health facilities, it allows users to examine gaps and duplication in service provision, assess the coverage of emergency services and the availability of doctors in a 24-hour period, calculate the shortest distance to referral facilities from any location, and determine whether a given facility is licensed and registered. This information is particularly useful in helping healthcare planners and policy makers make informed decisions around the distribution and monitoring of healthcare facilities and services,

For peer review only - http://bmjopen.bmj.com/site/about/guidelines.xhtml

and health human resources. For the general public, the tool holds promise in locating a desired healthcare service that is closest in distance, and indicating the shortest path to get there.

A key strength of this dataset is its inclusion of private-for-profit healthcare facilities, from pharmacies to hospitals, in addition to public and private not-for-profit healthcare provision. The UHA prototype has been demonstrated both nationally and internationally, and generated a great deal of interest and useful feedback. In Bangladesh, its promise has intrigued multiple stakeholder groups, ranging from the Directorate General of Health Services (DGHS) responsible for national healthcare planning, local government officials, private not-for-profit or non-government organizations (NGOs), service providers and development partners. In the context of significant investments in urban health systems strengthening that are in pipeline, and absence of urban data in the country's national health information system (DHIS2), UHA is widely regarded as timely and useful in the context of current urban health planning processes, and many discussions about its formal linkage to and institutionalization within existing health information systems have occurred.

However, due to the complexity of these kind of data, they risk being underutilized for health policy and planning unless specific efforts are attempted to make them more accessible to non-technical, policy and other local level stakeholders.[22] In the context of UHA, these efforts have included making the data available on the DGHS webpage, and organizing dissemination events in city corporations and municipalities. However, beyond anecdotal reports, there is no systematic information on whether the tool is being used by stakeholders, and how it could be improved to better meet their needs. The purpose of this study, therefore, is to pilot and refine the UHA for use in service delivery planning and referral, and by generating evidence on its utility,

For peer review only - http://bmjopen.bmj.com/site/about/guidelines.xhtml

inform and strengthen advocacy for and action around its institutionalization into the government system. A focus on MNCH service delivery was chosen to circumscribe the development of training materials, and to clearly delimit the range of stakeholders that should be engaged.

Study Aims

Three specific aims are identified in seeking to institutionalize the UHA for MNCH service delivery, planning, and referral into the government system:

1. To document stakeholder perceptions and experiences in adopting a tool that enables use of health facility information for strategic planning, day-to-day decision-making, control and oversight, and improved administrative efficiency of urban MNCH services.
2. To identify policy and programmatic entry points that will facilitate broader use of geo-referenced health facility information and its regular update.
3. To estimate costs associated with bringing geo-referenced facility listing into the government system

METHODS

This Implementation Research (IR) focuses on the factors and processes that influencing uptake, use and scale-up of ICT tools like UHA. The study will explore barriers in usability, understandability, and utility, as well as policy and other requirements needed to support its systematic implementation in the real world setting of healthcare planning, referral and oversight. The primary audiences of this research are managers and decision makers in the urban healthcare sector of Bangladesh.

Study design and participants

For peer review only - http://bmjopen.bmj.com/site/about/guidelines.xhtml

The proposed implementation research employs a mixed method research approach. Mixed-method research is a widely used approach in IR.[23] We will assess the uptake of UHA by the MNCH-related planners and decision makers over a 3-year period from 2016-2019. The specific IR variables to be assessed are adoption, appropriateness, feasibility, and implementation cost [23]. Many IR frameworks exist, however it is advised to use a framework befitting program parameters [23]. For this reason, we identified a toolkit specific to the development and use of ICT tools and formulated our conceptual framework accordingly. This helped to operationalize the research as an ICT intervention versus a clinical or health service level intervention, while still retaining some of the features of common IR frameworks, including concerns with guided implementation and innovation, sustainability, and stakeholder input.[23]

The WHO PATH toolkit was published by WHO and PATH to guide the introduction and implementation of information and communications technology (ICT) in health information systems.[18] The introduction of a new ICT tool is commonly accompanied by challenges that must be overcome. Before scaling-up, therefore, it is important to conduct rigorous product planning and feasibility testing, and to identify and engage key stakeholders. The toolkit identifies three main phases of an ICT project: pilot, scale, and sustain. In the pilot phase, the phase addressed in this study, a solution (UHA) is developed based on program needs and priorities, and tested on a small-scale to measure outcomes, impacts, and costs, and identify potential improvements. Several other factors influence the introduction of the HMIS in developing countries including planning, stakeholder roles and responsibilities, cultural aspects, human capacity, financial aspects sustainability etc. (see figure 1).[24] The elements of this ICT-informed framework are similar to that of the different phases of the “replicating effective programs framework” used in IR.[25] For instance, stakeholder needs in our framework are

For peer review only - http://bmjopen.bmj.com/site/about/guidelines.xhtml

addressed under the identifying implementation barriers step (pre-conditions phase), the orientation step of the pre-implementation phase is similar to participation and awareness and financial aspect elements in our framework, and training and technical assistance of the implementation phases are addressed through the human capacity development component.

In order to explore user perceptions, and policy and programmatic entry points, the policy engagement framework[22] also will be employed to prospectively analyze policy that incorporates strategies for change. This framework will confer a systematic approach to the ongoing collection, analysis and use of political information (e.g. concerning actors, their interests, institutions, ideas, and policy processes and context) that can alter the balance of power between those in support of and those resisting change by enabling pro-reformers to intervene more effectively in the policy process.[22]

Study sites

Two city corporations (CCs) – namely Dhaka North City Corporation (DNCC) and Dhaka South City Corporation (DSCC) – and two municipalities, Jessore and Dinajpur, which have geo-referenced health facility data available from previous mapping exercise of icddr,b[21] will be purposively selected. As smaller cities, Jessore and Dinajpur municipalities present marked differences from CCs in terms of size, structure, capacity and challenges posed.

Sample size

Through the stakeholder mapping, key urban MNCH decision-making actors at both national and local levels will be engaged to help identify potential users for UHA. This group will constitute

For peer review only - http://bmjopen.bmj.com/site/about/guidelines.xhtml

our study respondents and training participants. When determining sample size for qualitative research, Guest et al. propose that a homogenous group of respondents 12 interviews is sufficient for reaching data saturation.[26] It is also asserted that a sample under 20 respondents allows qualitative researchers to establish and maintain effective relationships with study participants, and thus enhances the validity of the research.[27] For these reasons, we will sample 15-16 respondents for each of our activities. The sampling strategy, type and number of respondents for each study activity are provided in table 1.

Table 1 Sampling strategy and sample type for each activity of the study

**Activity and focus**	**Data collection methods**	**Sampling strategy**	**Respondent group**	**Sample size**
**Task Review** (document how MNCH planning and referral decisions are currently made)	KIIs	Opportunistic/ Emergent sampling; Snowball sampling	–Urban health systems actors<br>–National and local government officials<br>–NGO programme managers	16
	Desk review	N/A	N/A	N/A

For peer review only

**User Need and User Experience** (explore user preferences, task needs and experiences)	IDIs (on user needs)	Opportunistic/ Emergent sampling; Snowball sampling	–Policy makers within the MOHFW<br>–Members of the Urban Health Cell of MOLGRDC<br>–Managers at City Corporations<br>–NGO programme managers	16
**Policy Engagement** (understand interactions between content, context, actors and processes of policy advocacy and entry points for sustainable incorporation of ICT into health systems)	KIIs	Opportunistic/ emergent sampling; Snowball sampling	–Urban health systems actors<br>–National and local government officials<br>–NGO programme managers	15
	Desk review (urban health and ICT related policies)	N/A	N/A	N/A

For peer review only - http://bmjopen.bmj.com/site/about/guidelines.xhtml

ICT, Information Communication and Technology; IDI, In-depth Interviews; KII, Key Informant Interviews; MNCH, Maternal, newborn and child health; MOHFW, Ministry of Health and Family Welfare; MOLGRDC, Ministry of Local Government, Rural Development and Cooperatives.

Implementation procedure of the pilot

The study implementation is envisaged to begin with stakeholder sensitization and partnership-building with government. This is critical because the research will work closely with the government health system in urban areas. In addition, an intervention will be conducted consisting of capacity building sessions around the use of the UHA for government and other urban health planners and managers. Details of these activities are provided below.

Stakeholder consultation and engagement: Two stakeholder consultation workshops will be carried out to identify and engage key stakeholders to create research buy-in and to begin the process of UHA advocacy. Detailed information on identification and mapping of stakeholders has been described in the data collection section. Ideas will be generated for uptake, regular use, and update of UHA.

Partnership development with Government: Implementation partnerships with the Management Information System (MIS) of the DGHS, MOHFW will be developed and a Memorandum of Understanding will be signed between icddr,b and DGHS. Permission letters will also be obtained from mayors of CCs and municipalities.

For peer review only - http://bmjopen.bmj.com/site/about/guidelines.xhtml

Development of training materials: A training manual on Urban Health Atlas (UHA) will be prepared to guide UHA capacity building workshops including case studies, guidelines for group work and hand-on activities, pre-test/post-test questionnaires, etc.

UHA workshops: In each study site, a 2-day UHA workshop, and subsequent 1 day refresher course, will be organized with a selected group of health workers and managers drawn from local government, and NGOs. Institutional agreements and permissions will be sought in advance from local government institutions and the health ministry as appropriate. Training sessions will provide an introduction to current urban health challenges, followed by an overview and demonstration of the Urban Health Atlas and its functions. Hands on training, group work and case studies will be undertaken to familiarize users with UHA and to get their feedback on how it might be improved to better meet their needs.

Data collection

Interviews will be conducted by an experienced group of researchers trained in qualitative interviewing including a mixed method expert, two software programmers, one GIS expert and an economist. The team will begin data collection in Dhaka, then move to the municipalities. A period of rapport building with key stakeholders in each study site will be critical to the success of this research given known difficulties in accessing the Government sector. Utilizing existing networks and negotiation skills will be especially important in opening doors and initiating discussion. The UHA tool will be assessed for impact on MNCH decision-making and outcomes. It is unlikely, however, that these effects would be apparent within three years of implementation. Thus, success of the tool will be determined based on user experiences as

For peer review only - http://bmjopen.bmj.com/site/about/guidelines.xhtml

specified by WHO PATH Toolkit i.e. better indicators for strategic planning, day-to-day decision-making, control and oversight, and reduced administrative burden.

Data collection methods for objective 1

To address objective 1, assessment of information needs and task review analysis will be done by desk review, in-depth interviews and click streams. Guidelines for qualitative interviews will be developed based on the WHO PATH toolkit's questions to measure success. Three qualitative research activities are envisaged to address this objective:

1. Key informant interviews (KIIs) with urban health system actors along with desk reviews to understand and document how MNCH planning and referral decisions are currently made.
2. In-depth interviews (IDIs) with potential UHA users to explore user preferences and task needs to refine the tool in advance of training.
3. IDIs with UHA users to understand their experiences and to document challenges and successes of using UHA for MNCH service decision-making during training and one- & three-months post-training.

In addition to qualitative assessments of user experiences, quantitative assessments of how different stakeholders are using data remotely will be made through (i) User's click streams; (ii) Task time devoted to different applications. Written feedback through online tools (i.e. Google Analytics) that facilitate remote testing will also be collected to generate more user-friendly functions that meet user needs.

For peer review only - http://bmjopen.bmj.com/site/about/guidelines.xhtml

Data collection methods for objective 2

To identify entry points that will facilitate broader use of geo-referenced facility information and its regular update, stakeholder mapping, policy mapping using desk review, KIIs, and stakeholder consultation workshop will be conducted. Guidelines for qualitative interviews will be developed using the policy engagement framework as a guide. Policy and programmatic entry points for the broader use and update of facility information, stakeholder analysis will be undertaken using Policy Engagement Framework as a guide. Stakeholders are identified as persons, groups, organization members or systems that affect or can be affected by a project/program/activity. Stakeholder analysis is an approach for generating knowledge about roles, behavior, inter-relation and intention of associated actors and their influence in implementation processes of a program or policy.[28] Given the importance of stakeholder satisfaction and support for the success of any program,[29,30] incorporating stakeholders' perspectives and needs is a critical step in gaining ownership around an ICT innovation like UHA and its incorporation into routine information systems, and use for decision-making. The following qualitative methods will be used to fulfill objective 2:

1. Stakeholder mapping including the identification and listing of stakeholder groups involved in urban health based on available literature and expert opinion.
2. A semi-structured guideline will be used to collect information during stakeholder consultation workshops to explore their respective interests, roles and responsibilities in urban health, their information needs, and perceptions of how they can contribute to institutionalizing UHA.

For peer review only - http://bmjopen.bmj.com/site/about/guidelines.xhtml

3. KIIs along with desk reviews will be undertaken to understand the processes of current health policy-making mechanisms and what other policies affect the integration of ICT in health.

Data collection methods for objective 3

To estimate cost of bringing facility listing into the government information system the total cost of ownership for UHA development and implementation will be estimated using an ingredient approach. Data will be collected through structured questionnaire, document review and KII. Cost will be estimated based on supply side aspects. The budget matrix will be developed with cost drivers proposed in the WHO PATH toolkit.

The cost for development and implementation of Urban Health Atlas (UHA) tools, coordination, and engagement of city corporations and DGHS will be estimated including both financial costs and economic costs of the program. Financial costs represent the actual expenditures on goods and services purchased. Economic costs include the estimated value of goods or services for which either there are no financial transactions or the price of a specific good did not reflect the cost of using it productively elsewhere.[31] The cost will be separated for start-up cost and implementation cost. The implementation cost comprises the costs required to run and maintain the ICT tools while executing intervention.[32]

All supply-side inputs will be identified, quantified and valued through a facility-level inventory, record reviews and key informant interviews. Both fixed cost and variable cost will be captured. Shared cost items, including salary, buildings, furniture, supervision, transportation, and vehicles, will be identified through observation and interviews of relevant personnel. Shared

For peer review only - http://bmjopen.bmj.com/site/about/guidelines.xhtml

costs will be apportioned by proportion of the time-involvement of the relevant items (i.e. office rent, common vehicle) to different activities. The time of volunteers will be transformed into costs by using the minimum wage level of manual workers in Bangladesh. There are some examples of components included in ingredient approaches (table 2):[32,33]

Table 2 Components included in ingredient approaches for cost

Method Name	Methodology	Inputs	Activities
Ingredients approach	Quantities x price, personnel, percentage use	Personnel, ICT tools, vehicles	Personnel , ICT tools, vehicles

ICT, Information Communication and Technology.

To identify activities of the UHA tool implementation and their related inputs, a review of program documents and interviews with relevant personnel will be conducted. The unit price/salary information will be collected from responsible program management. In case of missing unit price of any items, the market price of those items will be collected. Semi-structured checklists for cost data collection will be developed considering the program context and using WHO PATH toolkit as a guiding framework. The budget matrix will be completed with help from key personnel associated with costing and budgeting identified during the stakeholder mapping and research team's own estimates.

Data analysis

For peer review only - http://bmjopen.bmj.com/site/about/guidelines.xhtml

A process flowchart for current decision-making practices will be prepared using the KII and Organizational process reviews. A list of user needs will be made and shared at a stakeholder consultation meeting to identify the most important and feasible functions to be added to UHA.

For *qualitative data*, an outline plan for data analysis will be prepared in advance of research along with a priori codes. These codes, mostly focusing on user experience, will be derived from the WHO PATH toolkit.[18] The analysis will be open to emerging themes as well. All interviews will be recorded provided consent has been obtained, but with simultaneous note taking in case of equipment failure. Data transcription will occur immediately following each interview, followed by translation. Data familiarization will involve reading transcripts repeatedly to surface emerging themes and identify any missed opportunities for further exploration. Transcripts will be coded using ATLAS-ti (version-7.5.7). A team approach to analysis will be employed to minimize individual biases. Inter-coder reliability will be checked. Group discussions of emerging themes and patterns in the data will be tested using data displays that allow more systematic pattern-testing across respondents.

For *stakeholder analysis*, stakeholders' influence, importance and agreement will be explored applying standard techniques and measurement scales mentioned in table 3**.** A position diagram with level of agreement and level of influence will also be plotted to identify stakeholders who are already committed to work and help institutionalize UHA and those who need to be brought into agreement.

For peer review only - http://bmjopen.bmj.com/site/about/guidelines.xhtml

Table 3 Operational definitions for stakeholder analysis for policy engagement

Theme	Terms used	Operational definition
**Influence, importance and agreement analysis of stakeholders**	Level of influence	Stakeholders' influence will be determined according to each stakeholder group's perception and views on who is important in terms of urban health care delivery.
	Level of agreement	Stakeholders' agreement will be determined according to how much each stakeholder agreed.
	Level of importance	The stakeholders' importance will be determined according to how important each stakeholder group is to the other groups.
**Power and leadership**	Overall power	Power of a stakeholder-group will be assessed as compared to all other groups in Bangladeshi urban healthcare delivery system. Power of stakeholders will be measured as the product of multiplication of influence and importance.
	Relative position	Relative position of each stakeholder group will be assessed by comparing one group's position to other groups in broader scenario.
**Relative positions of**	Drivers	Stakeholders who have high level of importance as well as high level of influence on public sector health care delivery system

For peer review only - http://bmjopen.bmj.com/site/about/guidelines.xhtml

**stakeholders**	Supporters	Stakeholders who have high level of importance but low level of influence on urban healthcare delivery system
	Bystanders	Stakeholders who have low level of importance and low level of influence on urban healthcare delivery system
	Abstainers	Stakeholders who have no influence and no importance on urban healthcare delivery system
	Blockers	Stakeholders who have low level of importance but high level of influence on urban healthcare care delivery system.

For the *policy engagement analysis,* KIIs with stakeholders will be examined to understand interactions between actors, content, context and processes with respect to ICT policy uptake, with a view to identifying entry points for policy advocacy and the sustainable incorporation of ICT in health systems. Interviews will be analyzed using a priori codes drawn from the Policy Engagement Framework. Of additional interest in this analysis is understanding the mechanisms and processes of health policy development and how other policies may be important in efforts to integrate ICT into health systems. Based on these insights, a Health Policy for ICT process flowchart will be prepared. According to the policy engagement framework we will also seek information on how context is considered and dealt with when policies are formed and what processes need to be changed to more effectively integrate UHA into the system.[22]

For peer review only - http://bmjopen.bmj.com/site/about/guidelines.xhtml

For *quantitative data*, simple descriptive analysis will be performed to show user rates over time. The set of parameters to be analyzed are: number of users who accessed UHA, type of user, scope of UHA use, and types of problems faced. For data analysis, software like MS Excel and STATA will be used as appropriate.

The *cost* of implementing UHA will be estimated using a direct approach. Average cost for each activity will be calculated. All supply-side inputs will be identified, quantified and valued through record review and KIIs. Shared cost items, including salary, buildings, furniture, supervision, transportation, and vehicles will be identified through observation and interviews of relevant personnel. By considering the nature of inputs, these will be categorized into capital, recurrent as well as fixed and variable cost items. Shared costs will be apportioned by proportion of the time-involvement of the relevant items (office rent, common vehicle) to different activities. Inputs will be identified using discussion with relevant personnel, observation and record review. If unavailable, market prices will be applied to estimate costs. The capital items will be annualized, and common costs will be apportioned as per requirement. Annual values of capital items will be estimated from their expected useful life years and annuitization will be done using 3% discounting rate whenever applicable.[34] The study will allocate the cost for shared items (e.g. office space, appliances) by using actual utilization of items for this activity. Utilization information for shared items will be collected from responsible project staff.

Finally, the cost for shared items will be estimated by multiplying percentage-use information with the total cost of the items. Total cost will be calculated by summing up the start-up and

For peer review only - http://bmjopen.bmj.com/site/about/guidelines.xhtml

implementation costs. Relative contribution of start-up and implementation cost will be calculated. The cost-drivers in each activity will be identified considering the larger share of total cost.

Process documentation

In addition to all these activities, the study investigators will systematically document implementation processes for policy uptake and institutionalization of UHA, focusing particularly on contextual factors and their influence on implementation using a process documentation template. Process documentation of this "pilot phase" of UHA institutionalization will generate supporting knowledge to be applied the phases of scale-up and sustain, as specified in the WHO/PATH toolkit.

Patient and public involvement

There will be no direct patient or public involvement in this implementation research. However, a Technical Advisory Group (TAG) will be formulated for project governance, which will consist of representatives from Government, development partners, NGOs, academicians and senior researchers and urban health actors. Regular meetings will be held with partners and staff for problem solving. At the end of the study, the TAG will comment on the study findings and contribute to the dissemination plan.

Ethics

This study has received approval from the Research Review Committee (RRC) and the Ethical Review Committee (ERC) of icddr,b, both of which provided a thorough and critical review of

For peer review only - http://bmjopen.bmj.com/site/about/guidelines.xhtml

the protocol's technical and ethical aspects. Participants will be asked for written consent prior interviewing and will remain anonymous and unidentifiable. Tape recorders will be used to record discussions bur only after obtaining consent. All other form of data will be kept in locked storage, or controlled access folders, allowing only investigators of the study and members of the ERC of icddr,b to access information, if needed.

Dissemination

Findings from this research will be disseminated at various levels to develop interest and support from a wide variety of audiences i.e. public, private, NGO, civil society and donors. In doing so, we hope to build a diverse constituency of individuals and organizations willing and able to translate evidence yielded by the study, into policy action.

Local dissemination: Findings will be presented to relevant local administrators, development partners and NGOs and other relevant parties (local health practitioners), researchers.

National dissemination: A series of interactive workshops and briefing sessions with various stakeholders will be arranged to create linkages with national fora. The main aim will be to translate findings in a more visual and engaging format; i.e. research briefs and interactive project brochures, to reach a range of stakeholders.

International dissemination: This will include publishing findings in peer-reviewed journals and presenting in scientific forums, conferences and symposiums, and linking with international learning platforms. The main objective is to contribute to global knowledge about context

For peer review only - http://bmjopen.bmj.com/site/about/guidelines.xhtml

For peer review only

specific strategies to incorporate ICTs into health systems, and challenges that must be anticipated.

CONCLUSIONS

The proposed study seeks to understand the utility and processes by which the visualization of geo-referenced health facility information can contribute towards improving MNCH services in terms of strategy development, decision-making and oversight, with a special focus on the urban poor, and how best to approach the existing system for its institutionalization. The results from this study will inform efforts to scale-up the generation and application of facility listing data in urban areas nationwide. Beyond Bangladesh, this study is also expected to provide valuable guidance on how to generate user buy-in, and policy uptake necessary to introduce, scale-up, and sustain MNCH-related ICTs in similar LMICs. Once UHA becomes institutionalized, future research plans include evaluation of the use of the UHA and its impact on maternal and child health outcomes in urban areas in Bangladesh through longitudinal studies.

For peer review only

REFERENCES

1. Adams AM, Ahmed SM, Evans TG. Universal health care in Bangladesh–promises and perils. *Lancet Glob Health* 2018;6:e10-11.
2. Ahmed SM, Islam FK, Bhuiya A, ed. *Bangladesh Health Watch Report 2014: Urban Health Scenario: Looking Beyond 2015.* Dhaka: James P Grant School of Public Health, BRAC University, 2015.
3. Chowdhury AMR, Bhuiya A, Chowdhury ME, *et al*. The Bangladesh paradox: exceptional health achievement despite economic poverty. *Lancet* 2013;382:1734-45.
4. National Institute of Population Research and Training. *Bangladesh Urban Health Survey 2013; Final Report*. Dhaka: 4. National Institute of Population Research and Training, 2013.
5. National Institute of Population Research and Training. *Bangladesh Demographic and Health Survey 2014: Key Indicators*. Dhaka: 4. National Institute of Population Research and Training, 2016.
6. United Nations. Department of Economic and Social Affairs Population Dynamics.. World Urbanization Prospects2018. Available: https://esa.un.org/unpd/wup/publications/Files/WUP2018-KeyFacts.pdf
7. Alam SMN. Health service delivery: the state of government-non-government relations in Bangladesh. *Public Adm Dev* 2011;31:273-81.
8. Afsana K, Wahid SS. Health care for poor people in the urban slums of Bangladesh. *Lancet* 2013;382:2049-51.
9. Huque R, Barkat A, Sabina N. Public health expenditure: equity, efficacy and universal health coverage. *In*: Ahmed SM, Mahmud S, Evans TG, *et al*. ed. *Bangladesh Health*

For peer review only - http://bmjopen.bmj.com/site/about/guidelines.xhtml

Watch Report 2011: Moving towards Universal Health Coverage. Dhaka: James P Grant School of Public Health, BRAC University, 2012:25-32.

10. Osman F. Public health, urban governance and the poor in Bangladesh: policy and practice. *Asia-Pacific Dev J* 2009;16:27-58.
11. Matthews Z, Channon A, Neal S, Osrin D, Madise N, Stones W. Examining the "urban advantage" in maternal health care in developing countries. *PLoS Med* 2010;7:e1000327.
12. Local Government Division, Government of the People's Republic of Bangladesh. Urban Primary Health Care Services Delivery Project. Available: https://www.adb.org/projects/42177-024/main
13. Nutley T, Reynolds HW. Improving the Use of Health Data for Health System Strengthening. *Glob Health Action* 2013;6.
14. Few S. Data Visualization for Human Perception. In: *The Encyclopedia of Human – Computer Interaction*, 2nd Ed. Available: https://www.interaction-design.org/literature/book/the-encyclopedia-of-human-computer-interaction-2nd-ed/data-visualization-for-human-perception
15. World Health Organization. *Creating a Master Health Facility List*. Geneva: World Health Organization, 2013.
16. Rose-Wood A, Heard N, Thermidor R, *et al*. Development and use of a master health facility list: Haiti's experience during the 2010 earthquake response. *Glob Health Sci Pract* 2014;2:357-65.
17. World Health Organization. *Toolkit on monitoring health systems strengthening Measuring Health Systems Strengthening and Trends: A Toolkit for Countries*. Geneva: World Health Organization, 2008. Available at:

For peer review only

For peer review only - http://bmjopen.bmj.com/site/about/guidelines.xhtml

https://www.who.int/healthinfo/statistics/toolkit_hss/EN_PDF_Toolkit_HSS_Introduction.pdf

18. World Health Organization/PATH. *Planning an Information Systems Project: A Toolkit for Public Health Managers*. Seattle: PATH 2013.
19. World Health Organization. Master facility list resource package: guidance for countries wanting to strengthen their master facility list. Available at: https://www.who.int/healthinfo/country_monitoring_evaluation/mfl/en/
20. Adams AM, Ahmed S., Hasan SM,. *et al*. *Mapping the Urban Healthcare Landscape in 5 City Corporations, Bangladesh*. Dhaka: icddr,b, 2015.
21. icddrb. The Urban Health Atlas. Available: http://www.urbanhealthatlas.com
22. Buse K, Booth D, Murindwa G, *et al*. *Donors and the Political Dimensions of Health Sector Reform: The Cases of Tanzania and Uganda*. London: Overseas Development Institute, 2008.
23. Peters DH, Adam T, Alonge O, Agyepong IA, Tran N. Implementation research: what it is and how to do it. *BMJ* 2013;347:f6753.
24. Archangel N. *The critical issues affecting the introduction of Health Management Information Systems in developing countries in Africa*. Amsterdam: Universiteit van Amsterdam, 2007.
25. Kilbourne AM, Neumann MS, Pincus HA, Bauer MS, Stall R. Implementing evidence-based interventions in health care: application of the replicating effective programs framework. *Implement Sci.* 2007;2:42.
26. Guest G, Bunce A, Johnson L. How many interviews are enough? An experiment with data saturation and variability. *Field Methods.* 2006;18:59-82.

For peer review only - http://bmjopen.bmj.com/site/about/guidelines.xhtml

For peer review only

27. Crouch M, McKenzie H. The logic of small samples in interview-based qualitative research. *Soc Sci Inf.* 2006;45:483-99.

28. Bryson JM. What to do when stakeholders matter: stakeholder identification and analysis techniques. *Public Manag Rev*. 2004;6:21-53.

29. Bryson JM. *Strategic planning for public and nonprofit organizations: A guide to strengthening and sustaining organizational achievementVol 1.* San Francisco, CA: Jossey-Bass Publishers, 1995.

30. Moore MH. *Creating public value: Strategic management in government*. Cambridge, MA: Harvard University Press, 1995.

31. Gold MR, Siegel JE, Russell LB, *et al*. ed. *Cost-effectiveness in Health and Medicine 1ed*. New York: Oxford University Press, 1996.

32. Drummond MF, Sculpher MJ, Claxton K, *et al*. *Methods for the economic evaluation of health care programmes*. Oxford: Oxford University Press 2015.

33. World Health Organization. *Comprehensive Multi-Year Planning (cMYP): A Tool and User Guide for cMYP Costing and Financing*. Geneva:World Health Organization, 2014.

34. Fiedler JL, Villalobos CA, De Mattos AC. An activity-based cost analysis of the Honduras community-based, integrated child care (AIN-C) programme. *Health Policy Plan* 2008;23:408-27.

For peer review only - http://bmjopen.bmj.com/site/about/guidelines.xhtml

Figure 1. Modified conceptual framework for Information Communication and Technology (ICT) implementation in developing countries

For peer review only

For peer review only - http://bmjopen.bmj.com/site/about/guidelines.xhtml

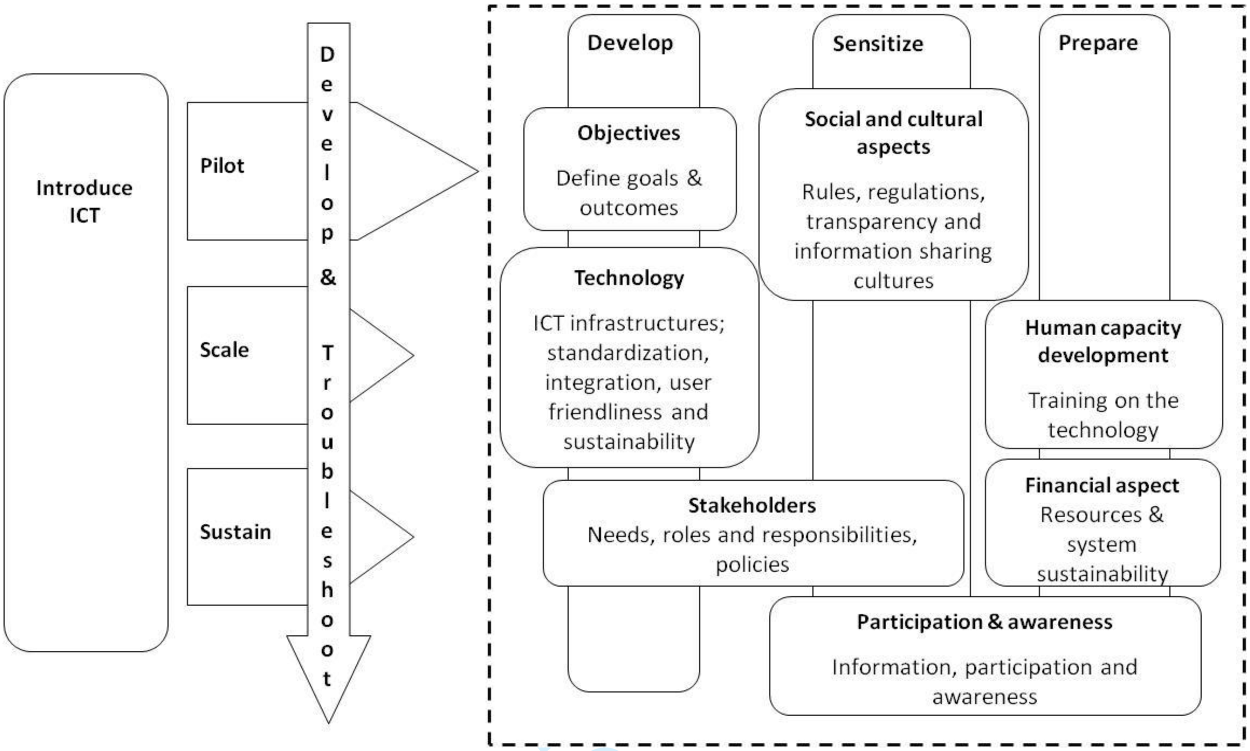

Figure 1. Modified Conceptual framework for Information Communication and Technology (ICT) implementation in developing countries

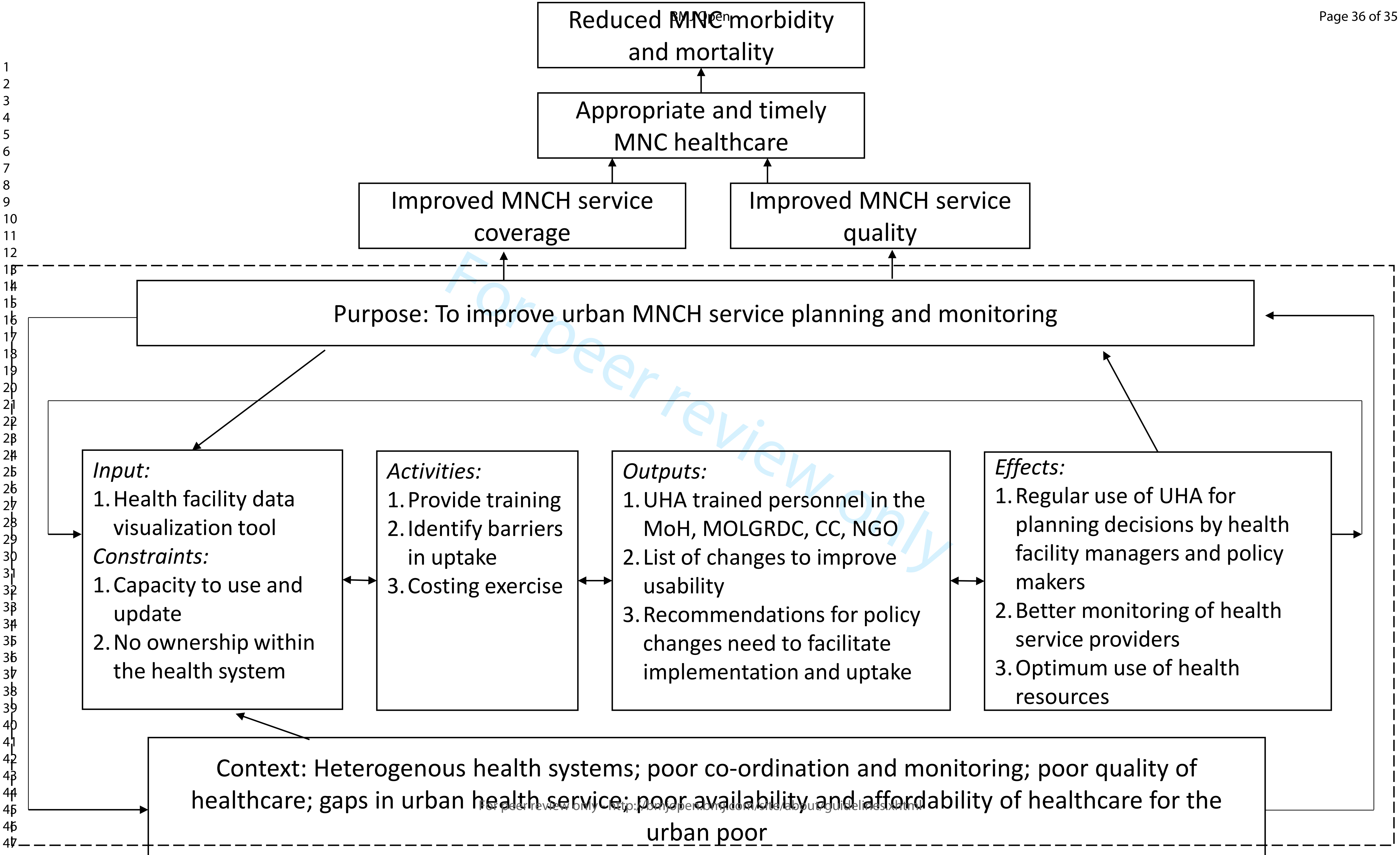

For peer review only

BMJ Open

Making Information and Communications Technologies (ICTs) work for health: protocol for a mixed-methods study exploring processes for institutionalizing geo-referenced health information systems to strengthen Maternal Neonatal and Child Health (MNCH) service planning, referral and oversight in urban Bangladesh

Journal:	*BMJ Open*
Manuscript ID	bmjopen-2019-032820.R2
Article Type:	Protocol
Date Submitted by the Author:	20-Jun-2020

Complete List of Authors:	Islam, Rubana ; University of New South Wales, School of Public Health and Community Medicine<br>Adams, Alayne; McGill University, Department of Family Medicine; BRAC University James P Grant School of Public Health<br>Hasan, Shaikh Mehdi ; International Centre for Diarrhoeal Disease Research Bangladesh, Health Systems and Population Studies Division<br>AHMED, RUSHDIA; International Centre for Diarrhoeal Disease Research Bangladesh, Health Systems and Population Studies Division<br>Bhattacharyya, Dipika Shankar; International Centre for Diarrhoeal Disease Research, Health Systems and Population Studies Division<br>Shafique, Sohana; International Centre for Diarrhoeal Disease Research, Health Systems and Population Studies Division

**Primary Subject Heading**:	Public health
Secondary Subject Heading:	Global health, Health policy, Health informatics, Health services research
Keywords:	Urban health, maternal, neonatal and child health, information communication and technology, geographic information systems, health management information systems, Bangladesh

SCHOLARONE™
Manuscripts

I, the Submitting Author has the right to grant and does grant on behalf of all authors of the Work (as defined in the below author licence), an exclusive licence and/or a non-exclusive licence for contributions from authors who are: i) UK Crown employees; ii) where BMJ has agreed a CC-BY licence shall apply, and/or iii) in accordance with the terms applicable for US Federal Government officers or employees acting as part of their official duties; on a worldwide, perpetual, irrevocable, royalty-free basis to BMJ Publishing Group Ltd ("BMJ") its licensees and where the relevant Journal is co-owned by BMJ to the co-owners of the Journal, to publish the Work in this journal and any other BMJ products and to exploit all rights, as set out in our licence.

The Submitting Author accepts and understands that any supply made under these terms is made by BMJ to the Submitting Author unless you are acting as an employee on behalf of your employer or a postgraduate student of an affiliated institution which is paying any applicable article publishing charge ("APC") for Open Access articles. Where the Submitting Author wishes to make the Work available on an Open Access basis (and intends to pay the relevant APC), the terms of reuse of such Open Access shall be governed by a Creative Commons licence – details of these licences and which Creative Commons licence will apply to this Work are set out in our licence referred to above.

Other than as permitted in any relevant BMJ Author's Self Archiving Policies, I confirm this Work has not been accepted for publication elsewhere, is not being considered for publication elsewhere and does not duplicate material already published. I confirm all authors consent to publication of this Work and authorise the granting of this licence.

For peer review only

Title: Making Information and Communications Technologies (ICTs) work for health: protocol for a mixed-methods study exploring processes for institutionalizing geo-referenced health information systems to strengthen Maternal Neonatal and Child Health (MNCH) service planning, referral and oversight in urban Bangladesh

Authors

Rubana Islam (rubana.islam@student.unsw.edu.au)[1*]

Alayne Adams (alayne.adams@gmail.com)[2*]

Shaikh Mehdi Hasan (smehdi@icddrb.org)[3]

Rushdia Ahmed (ahmed.rushdia@yahoo.com)[3]

Dipika Shankar Bhattacharyya (dipikashankar@gmail.com)[3]

Sohana Shafique (sohana.shafique@icddrb.org)[3**]

Author Affiliations

[1] School of Public Health and Community Medicine, University of New South Wales, Sydney, Australia

[2] Department of Family Medicine, Faculty of Medicine, McGill University, Montréal, Québec, Canada

[3] Universal Health Coverage Programme, Health Systems and Population Studies Division, icddr,b, Dhaka, Bangladesh

*These authors contributed equally for this work.

For peer review only

**Corresponding author/request for reprints:

Dr. Sohana Shafique

Assistant Scientist and Deputy Project Coordinator

Universal Health Coverage programme

Health Systems and Population Studies Division

icddr,b, Mohakhali, Dhaka. Bangladesh.

Email: sohana.shafique@icddrb.org

Phone: +880-2-9827001-10, ext. 2273

Word Count: 4550

Author Contributions

RI & AA conceptualized the study. RI & AA prepared the first draft of the manuscript. SMH, RA, DSB and SS revised the manuscript. RI & AA reviewed critically for important intellectual content; SS revised the version submitted with inputs from all other co-authors.

Acknowledgements

This research study was funded by International Development Research Centre (IDRC). icddr,b acknowledges with gratitude the commitment of IDRC to its research efforts. icddr,b is also grateful to the Governments of Bangladesh, Canada, Sweden and the UK for providing core/unrestricted support.

For peer review only - http://bmjopen.bmj.com/site/about/guidelines.xhtml

Competing interests

The authors declare no competing interests.

Funding

This work was supported by International Development Research Centre (IDRC), Canada (Grant Number: 108218-001).

Study period

2016-2019

Data statement section

Data will be handled according to the principles of the icddr,b policies and guidelines of the International Development Research Centre, Canada.

For peer review only

For peer review only - http://bmjopen.bmj.com/site/about/guidelines.xhtml

Abbreviations

ADB	Asian Development Bank
CCs	City Corporations
DGHS	Directorate General of Health Services
ERC	Ethical Review Committee
GIS	Geographic Information System
HMIS	Health Management Information System
ICT	Information Communication and Technology
IDI	In-depth Interviews
KII	Key Informant Interviews
LMICs	Low and Middle Income Countries
MNCH	Maternal, newborn and child health
MOHFW	Ministry of Health and Family Welfare
MOLGRDC	Ministry of Local Government, Rural Development and Cooperatives
NGOs	Non-Government Organizations
PHC	Primary Health Care
RRC	Research Review Committee
SDGs	Sustainable Development Goals
UHA	Urban Health Atlas
UHC	Universal Health Coverage
WHO	World Health Organization

For peer review only - http://bmjopen.bmj.com/site/about/guidelines.xhtml

ABSTRACT

Introduction: Disparities in health outcomes and access to maternal neonatal and child health (MNCH) are apparent among urban poor compared to national, rural or urban averages. A fundamental first step in addressing inequities in MNCH services is, knowing what services exist in urban areas, where these are located, who provides them, and who uses them. This study aims to institutionalize the Urban Health Atlas (UHA) - a novel ICT tool - to strengthen health service delivery and oversight and generate critical evidence to inform health policy and planning in urban Bangladesh.

Methods and analysis: This mixed-method implementation research will be conducted in four purposively selected urban sites representing larger and smaller cities. Research activities will include an assessment of information needs and task review analysis of information users, stakeholder mapping, and cost estimation. To document stakeholder perceptions and experiences, Key Informant Interviews (KIIs) and In-depth Interviews (IDIs) will be conducted along with desk reviews to understand MNCH planning and referral decisions. The UHA will be refined to increase responsiveness to user needs and capacities, and hands-on training will be provided to health managers. Cost estimation will be conducted to assess the financial implications of UHA uptake and scale-up. Systematic documentation of the implementation process will be undertaken. Policy decision-making and ICT health policy process flowcharts will be prepared using desk reviews and qualitative interviews. Thematic analysis of qualitative data will involve both emergent and a priori coding guided by WHO PATH toolkit and Policy Engagement Framework. Stakeholder analysis will apply standard techniques and measurement scales. Descriptive analysis of quantitative data and cost estimation analysis will also be performed.

For peer review only

Ethics and dissemination: The study has been approved by the Institutional Review Board of icddr,b (# PR–16057). Study findings will be disseminated through national and international workshops, conferences, policy briefs and peer-reviewed publications.

Keywords

Urban health, maternal, neonatal and child health, information communication and technology, geographic information systems, health management information systems, Bangladesh.

Article Summary

Strengths and limitations of this study

- This mixed method implementation research is among the first in Bangladesh to explore processes for institutionalizing geo-referenced health information systems to strengthen MNCH service planning, referral, and oversight in urban areas.
- The proposed research will generate knowledge to enhance understanding of how geo-referenced health facility information can inform MNCH service planning and decision making.
- This study is expected to provide valuable guidance beyond Bangladesh's HMIS on how to generate user buy-in and policy uptake necessary to introduce MNCH-related ICTs in developing country contexts.

For peer review only

For peer review only - http://bmjopen.bmj.com/site/about/guidelines.xhtml

INTRODUCTION

Bangladesh has embraced the Sustainable Development Goal-3 (SDGs) of achieving universal health coverage by 2030,[1] however, challenges related to rapid population growth, pluralistic health systems, and lack of governance, among others, are substantial.[2] Although Bangladesh has made extraordinary progress in reducing maternal and child mortality,[3] there are significant disparities in health-related outcomes and access to maternal neonatal and child health (MNCH) services stratified along both socioeconomic and geographic dimensions. Health indicators are far worse in urban slums than both non-slum urban areas and the national average.[4] Nationally, the mortality rate for children under five years of age is 65 per 1,000 live births and 49 per 1,000 live births in rural areas while the rate is 81 per 1000 live births among urban slum residents.[4, 5] Undergoing rapid urbanization, the country is projected to become over 50% urban by 2040, with almost one-third of urban residents living in slums.[6] Persistent inequities in key MNCH indicators in urban areas highlight the need to focus on issues of service coverage, access, quality and timely and appropriate referral as urgent policy priorities.

The Bangladesh urban health system is a smorgasbord of service providers characterized by inadequate coordination and regulation, and geographic and socioeconomic inequities in healthcare access.[7, 8] Several reasons have been proposed for inefficiencies in the system including poor planning and management capacity, weak coordination among the authorities, lack of clear, separate roles and responsibilities for the various authorities, service coverage gaps, and human resource management issues.[9, 10] Of particular concern in urban areas is the lack of adequate public primary care infrastructure and services which disproportionately impacts the urban poor, and poses significant challenges to the country’s aspirations to meet the goal of Universal Health Coverage by 2030.[11] One consequence of limited formal primary healthcare

For peer review only - http://bmjopen.bmj.com/site/about/guidelines.xhtml

services in urban areas[12] is the emergence of the private sector in health including the proliferation of informal providers such as pharmacies on which many of the urban poor rely. The formal private sector is equally massive, accounting for 80% of over 3500 hospitals in Bangladesh, and an even greater percentage in urban areas. Lack of regulation of this sector has resulted in concerns about quality of care and financial accessibility, especially for the urban poor.[10]

A fundamental first step in addressing inequities in urban healthcare access is an in-depth understanding of what services exist, their location, who provides them, and who utilizes them. A strong health management information system (HMIS), an essential component of sound programme development and implementation. Enabling the use of data for strategic decision-making, better governance, institutionalized HMIS systems represent the foundation upon which improvements in health outcomes can be monitored and greater accountability ensured.[13, 14] A Master Facility List (MFL) is a crucial constituent of HIS and permits the linkage of sub-systems within national HIS architecture.[15,16] MFL is advocated by The World Health Organization (WHO) as an effective means of ensuring better governance including systematic reporting and monitoring supervision.[17,18] MFLs like UHA are expected to facilitate health service planning and management through mapping or visualizing the distribution of health services and resources. It can also assist health service providers in identifying appropriate referral facilities for patients.[19] These functions can help improve equitable service coverage and reduce delays in receiving appropriate care, which in turn can impact health outcomes such as maternal and child mortality among the urban poor. A theory of change is provided in supplemental file 1.

For peer review only - http://bmjopen.bmj.com/site/about/guidelines.xhtml

Realizing the critical role that health information systems play in health management and building on political commitment towards "Digital Bangladesh", the Bangladesh Directorate General of Health Services (DGHS), Ministry of Health and Family Welfare (MOHFW) is implementing the District Health Information Software-2 (DHIS2) with support from development partners. While the system has been rolled out nationally, information is largely confined to public healthcare facilities. In urban areas, with the exception of large public hospitals and a number of NGOs involved in primary care provision, data are particularly sparse, especially for the massive private sector.

Urban Health Atlas: A novel ICT tool

Addressing this information gap, icddr,b has created a geo-referenced heath facility database for nine major cities and municipalities across Bangladesh. This dataset consists of a census of all healthcare facilities and the services they provide along with their geo locations.[20] To enable the practical application of this dataset, an Information Communication and Technology (ICT) tool called *Urban Health Atlas* (UHA) was developed (http://urbanhealthatlas.com).[21] This GIS-based interactive online tool displays health facility data visually and permits their manipulation for better healthcare planning and decision making. Providing detailed information on the location and services available at public and private health facilities, it allows users to examine gaps and duplication in service provision, assess the coverage of emergency services and the availability of doctors in a 24-hour period, calculate the shortest distance to referral facilities from any location, and determine whether a given facility is licensed and registered. This information is particularly useful in helping healthcare planners and policy makers make informed decisions around the distribution and monitoring of healthcare facilities and services,

For peer review only - http://bmjopen.bmj.com/site/about/guidelines.xhtml

and health human resources. For the general public, the tool holds promise in locating a desired healthcare service that is closest in distance and indicating the shortest path to get there.

A key strength of this dataset is its inclusion of private-for-profit healthcare facilities, from pharmacies to hospitals, in addition to public and private not-for-profit healthcare provision. The UHA prototype has been demonstrated both nationally and internationally, and generated a great deal of interest and useful feedback. In Bangladesh, its promise has intrigued multiple stakeholder groups, ranging from the Directorate General of Health Services (DGHS) responsible for national healthcare planning, local government officials, private not-for-profit or non-government organizations (NGOs), service providers and development partners. In the context of significant investments in urban health systems strengthening that are in pipeline, and absence of urban data in the country's national health information system (DHIS2), UHA is widely regarded as timely and useful in the context of current urban health planning processes, and many discussions about its formal linkage to and institutionalization within existing health information systems have occurred.

However, due to the complexity of these kind of data, they risk being underutilized for health policy and planning unless specific efforts are attempted to make them more accessible to non-technical, policy and other local level stakeholders.[22] In the context of UHA, these efforts have included making the data available on the DGHS webpage, and organizing dissemination events in city corporations and municipalities. However, beyond anecdotal reports, there is no systematic information on whether the tool is being used by stakeholders, and how it could be improved to better meet their needs. The purpose of this study, therefore, is to pilot and refine the UHA for use in service delivery planning and referral, and by generating evidence on its utility,

For peer review only - http://bmjopen.bmj.com/site/about/guidelines.xhtml

inform and strengthen advocacy for and action around its institutionalization into the government system. A focus on MNCH service delivery was chosen to circumscribe the development of training materials, and to clearly delimit the range of stakeholders that should be engaged.

Study Aims

Three specific aims are identified in seeking to institutionalize the UHA for MNCH service delivery, planning, and referral into the government system:

1. To document stakeholder perceptions and experiences in adopting a tool that enables use of health facility information for strategic planning, day-to-day decision-making, control and oversight, and improved administrative efficiency of urban MNCH services.
2. To identify policy and programmatic entry points that will facilitate broader use of geo-referenced health facility information and its regular update.
3. To estimate costs associated with bringing geo-referenced facility listing into the government system

METHODS

This Implementation Research (IR) focuses on the factors and processes that influencing uptake, use and scale-up of ICT tools like UHA. The study will explore barriers in usability, understandability, and utility, as well as policy and other requirements needed to support its systematic implementation in the real world setting of healthcare planning, referral, and oversight. The primary audiences of this research are managers and decision makers in the urban healthcare sector of Bangladesh.

Study design and participants

For peer review only - http://bmjopen.bmj.com/site/about/guidelines.xhtml

The proposed implementation research employs a mixed method research approach. Mixed-method research is a widely used approach in IR.[23] We will assess the uptake of UHA by the MNCH-related planners and decision makers over a 3-year period from 2016-2019. The specific IR variables to be assessed are adoption, appropriateness, feasibility, and implementation cost [23]. Many IR frameworks exist, however it is advised to use a framework befitting program parameters [23]. For this reason, we identified a toolkit specific to the development and use of ICT tools and formulated our conceptual framework accordingly. This helped to operationalize the research as an ICT intervention versus a clinical or health service level intervention, while still retaining some of the features of common IR frameworks, including concerns with guided implementation and innovation, sustainability, and stakeholder input.[23]

The WHO PATH toolkit was published by WHO and PATH to guide the introduction and implementation of information and communications technology (ICT) in health information systems.[18] The introduction of a new ICT tool is commonly accompanied by challenges that must be overcome. Before scaling-up, therefore, it is important to conduct rigorous product planning and feasibility testing, and to identify and engage key stakeholders. The toolkit identifies three main phases of an ICT project: pilot, scale, and sustain. In the pilot phase, the phase addressed in this study, a solution (UHA) is developed based on program needs and priorities, and tested on a small-scale to measure outcomes, impacts, and costs, and identify potential improvements. Several other factors influence the introduction of the HMIS in developing countries including planning, stakeholder roles and responsibilities, cultural aspects, human capacity, financial aspects sustainability etc. (see figure 1).[24] The elements of this ICT-informed framework are similar to that of the different phases of the “replicating effective programs framework” used in IR.[25] For instance, stakeholder needs in our framework are

For peer review only - http://bmjopen.bmj.com/site/about/guidelines.xhtml

addressed under the identifying implementation barriers step (pre-conditions phase), the orientation step of the pre-implementation phase is similar to participation and awareness and financial aspect elements in our framework, and training and technical assistance of the implementation phases are addressed through the human capacity development component.

In order to explore user perceptions, and policy and programmatic entry points, the policy engagement framework [22] also will be employed to prospectively analyze policy that incorporates strategies for change. This framework will confer a systematic approach to the ongoing collection, analysis and use of political information (e.g. concerning actors, their interests, institutions, ideas, and policy processes and context) that can alter the balance of power between those in support of and those resisting change by enabling pro-reformers to intervene more effectively in the policy process.[22]

Study sites

Two city corporations (CCs) – namely Dhaka North City Corporation (DNCC) and Dhaka South City Corporation (DSCC) – and two municipalities, Jessore and Dinajpur, which have geo-referenced health facility data available from previous mapping exercise of icddr,b[21] will be purposively selected. As smaller cities, Jessore and Dinajpur municipalities present marked differences from CCs in terms of size, structure, capacity, and challenges posed.

Sample size

Through the stakeholder mapping, key urban MNCH decision-making actors at both national and local levels will be engaged to help identify potential users for UHA. This group will constitute

For peer review only - http://bmjopen.bmj.com/site/about/guidelines.xhtml

our study respondents and training participants. When determining sample size for qualitative research, Guest et al. propose that a homogenous group of respondents 12 interviews is sufficient for reaching data saturation.[26] It is also asserted that a sample under 20 respondents allows qualitative researchers to establish and maintain effective relationships with study participants, and thus enhances the validity of the research.[27] For these reasons, we will sample 15-16 respondents for each of our activities. The sampling strategy, type, and number of respondents for each study activity are provided in table 1.

Table 1 Sampling strategy and sample type for each activity of the study

**Activity and focus**	**Data collection methods**	**Sampling strategy**	**Respondent group**	**Sample size**
**Task Review** (document how MNCH planning and referral decisions are currently made)	KIIs	Opportunistic/ Emergent sampling; Snowball sampling	–Urban health systems actors<br>–National and local government officials<br>–NGO programme managers	16
	Desk review	N/A	N/A	N/A

For peer review only

**User Need and User Experience** (explore user preferences, task needs and experiences)	IDIs (on user needs)	Opportunistic/ Emergent sampling; Snowball sampling	–Policy makers within the MOHFW<br>–Members of the Urban Health Cell of MOLGRDC<br>–Managers at City Corporations<br>–NGO programme managers	16
**Policy Engagement** (understand interactions between content, context, actors and processes of policy advocacy and entry points for sustainable incorporation of ICT into health systems)	KIIs	Opportunistic/ emergent sampling; Snowball sampling	–Urban health systems actors<br>–National and local government officials<br>–NGO programme managers	15
	Desk review (urban health and ICT related policies)	N/A	N/A	N/A

For peer review only - http://bmjopen.bmj.com/site/about/guidelines.xhtml

ICT, Information Communication and Technology; IDI, In-depth Interviews; KII, Key Informant Interviews; MNCH, Maternal, newborn and child health; MOHFW, Ministry of Health and Family Welfare; MOLGRDC, Ministry of Local Government, Rural Development and Cooperatives.

Implementation procedure of the pilot

The study implementation is envisaged to begin with stakeholder sensitization and partnership-building with government. This is critical because the research will work closely with the government health system in urban areas. In addition, an intervention will be conducted consisting of capacity building sessions around the use of the UHA for government and other urban health planners and managers. Details of these activities are provided below.

Stakeholder consultation and engagement: Two stakeholder consultation workshops will be carried out to identify and engage key stakeholders to create research buy-in and to begin the process of UHA advocacy. Detailed information on identification and mapping of stakeholders has been described in the data collection section. Ideas will be generated for uptake, regular use, and update of UHA.

Partnership development with Government: Implementation partnerships with the Management Information System (MIS) of the DGHS, MOHFW will be developed and a Memorandum of Understanding will be signed between icddr,b and DGHS. Permission letters will also be obtained from mayors of CCs and municipalities.

For peer review only - http://bmjopen.bmj.com/site/about/guidelines.xhtml

Development of training materials: A training manual on Urban Health Atlas (UHA) will be prepared to guide UHA capacity building workshops including case studies, guidelines for group work and hand-on activities, pre-test/post-test questionnaires, etc.

UHA workshops: In each study site, a 2-day UHA workshop, and subsequent 1-day refresher course, will be organized with a selected group of health workers and managers drawn from local government, and NGOs. Institutional agreements and permissions will be sought in advance from local government institutions and the health ministry as appropriate. Training sessions will provide an introduction to current urban health challenges, followed by an overview and demonstration of the Urban Health Atlas and its functions. Hands on training, group work and case studies will be undertaken to familiarize users with UHA and to get their feedback on how it might be improved to better meet their needs.

Data collection

Interviews will be conducted by an experienced group of researchers trained in qualitative interviewing including a mixed method expert, two software programmers, one GIS expert and an economist. The team will begin data collection in Dhaka, then move to the municipalities. A period of rapport building with key stakeholders in each study site will be critical to the success of this research given known difficulties in accessing the Government sector. Utilizing existing networks and negotiation skills will be especially important in opening doors and initiating discussion. The UHA tool will be assessed for impact on MNCH decision-making and outcomes. It is unlikely, however, that these effects would be apparent within three years of implementation. Thus, success of the tool will be determined based on user experiences as

For peer review only - http://bmjopen.bmj.com/site/about/guidelines.xhtml

specified by WHO PATH Toolkit i.e. better indicators for strategic planning, day-to-day decision-making, control and oversight, and reduced administrative burden.

Data collection methods for objective 1

To address objective 1, assessment of information needs and task review analysis will be done by desk review, in-depth interviews and click streams. Guidelines for qualitative interviews will be developed based on the WHO PATH toolkit's questions to measure success. Three qualitative research activities are envisaged to address this objective:

1. Key informant interviews (KIIs) with urban health system actors along with desk reviews to understand and document how MNCH planning and referral decisions are currently made.
2. In-depth interviews (IDIs) with potential UHA users to explore user preferences and task needs to refine the tool in advance of training.
3. IDIs with UHA users to understand their experiences and to document challenges and successes of using UHA for MNCH service decision-making during training and one- & three-months post-training.

In addition to qualitative assessments of user experiences, quantitative assessments of how different stakeholders are using data remotely will be made through (i) User's click streams; (ii) Task time devoted to different applications. Written feedback through online tools (i.e. Google Analytics) that facilitate remote testing will also be collected to generate more user-friendly functions that meet user needs.

For peer review only - http://bmjopen.bmj.com/site/about/guidelines.xhtml

Data collection methods for objective 2

To identify entry points that will facilitate broader use of geo-referenced facility information and its regular update, stakeholder mapping, policy mapping using desk review, KIIs, and stakeholder consultation workshop will be conducted. Guidelines for qualitative interviews will be developed using the policy engagement framework as a guide. Policy and programmatic entry points for the broader use and update of facility information, stakeholder analysis will be undertaken using Policy Engagement Framework as a guide. Stakeholders are identified as persons, groups, organization members or systems that affect or can be affected by a project/program/activity. Stakeholder analysis is an approach for generating knowledge about roles, behavior, inter-relation and intention of associated actors and their influence in implementation processes of a program or policy.[28] Given the importance of stakeholder satisfaction and support for the success of any program,[29,30] incorporating stakeholders' perspectives and needs is a critical step in gaining ownership around an ICT innovation like UHA and its incorporation into routine information systems, and use for decision-making. The following qualitative methods will be used to fulfill objective 2:

1. Stakeholder mapping including the identification and listing of stakeholder groups involved in urban health based on available literature and expert opinion.
2. A semi-structured guideline will be used to collect information during stakeholder consultation workshops to explore their respective interests, roles and responsibilities in urban health, their information needs, and perceptions of how they can contribute to institutionalizing UHA.

For peer review only - http://bmjopen.bmj.com/site/about/guidelines.xhtml

3. KIIs along with desk reviews will be undertaken to understand the processes of current health policy-making mechanisms and what other policies affect the integration of ICT in health.

Data collection methods for objective 3

To estimate cost of bringing facility listing into the government information system the total cost of ownership for UHA development and implementation will be estimated using an ingredient approach. Data will be collected through structured questionnaire, document review and KII. Cost will be estimated based on supply side aspects. The budget matrix will be developed with cost drivers proposed in the WHO PATH toolkit.

The cost for development and implementation of Urban Health Atlas (UHA) tools, coordination, and engagement of city corporations and DGHS will be estimated including both financial costs and economic costs of the program. Financial costs represent the actual expenditures on goods and services purchased. Economic costs include the estimated value of goods or services for which either there are no financial transactions or the price of a specific good did not reflect the cost of using it productively elsewhere.[31] The cost will be separated for start-up cost and implementation cost. The implementation cost comprises the costs required to run and maintain the ICT tools while executing intervention.[32]

All supply-side inputs will be identified, quantified, and valued through a facility-level inventory, record reviews and key informant interviews. Both fixed cost and variable cost will be captured. Shared cost items, including salary, buildings, furniture, supervision, transportation, and vehicles, will be identified through observation and interviews of relevant personnel. Shared

For peer review only - http://bmjopen.bmj.com/site/about/guidelines.xhtml

costs will be apportioned by proportion of the time-involvement of the relevant items (i.e. office rent, common vehicle) to different activities. The time of volunteers will be transformed into costs by using the minimum wage level of manual workers in Bangladesh. There are some examples of components included in ingredient approaches (table 2):[32,33]

Table 2 Components included in ingredient approaches for cost

Method Name	Methodology	Inputs	Activities
Ingredients approach	Quantities x price, personnel, percentage use	Personnel, ICT tools, vehicles	Personnel, ICT tools, vehicles

ICT, Information Communication and Technology.

To identify activities of the UHA tool implementation and their related inputs, a review of program documents and interviews with relevant personnel will be conducted. The unit price/salary information will be collected from responsible program management. In case of missing unit price of any items, the market price of those items will be collected. Semi-structured checklists for cost data collection will be developed considering the program context and using WHO PATH toolkit as a guiding framework. The budget matrix will be completed with help from key personnel associated with costing and budgeting identified during the stakeholder mapping and research team's own estimates.

Data analysis

For peer review only - http://bmjopen.bmj.com/site/about/guidelines.xhtml

A process flowchart for current decision-making practices will be prepared using the KII and Organizational process reviews. A list of user needs will be made and shared at a stakeholder consultation meeting to identify the most important and feasible functions to be added to UHA.

For *qualitative data*, an outline plan for data analysis will be prepared in advance of research along with a priori codes. These codes, mostly focusing on user experience, will be derived from the WHO PATH toolkit.[18] The analysis will be open to emerging themes as well. All interviews will be recorded provided consent has been obtained, but with simultaneous note taking in case of equipment failure. Data transcription will occur immediately following each interview, followed by translation. Data familiarization will involve reading transcripts repeatedly to surface emerging themes and identify any missed opportunities for further exploration. Transcripts will be coded using ATLAS-ti (version-7.5.7). A team approach to analysis will be employed to minimize individual biases. Inter-coder reliability will be checked. Group discussions of emerging themes and patterns in the data will be tested using data displays that allow more systematic pattern-testing across respondents.

For *stakeholder analysis*, stakeholders' influence, importance, and agreement will be explored applying standard techniques and measurement scales mentioned in table 3**.** A position diagram with level of agreement and level of influence will also be plotted to identify stakeholders who are already committed to work and help institutionalize UHA and those who need to be brought into agreement.

For peer review only

Table 3 Operational definitions for stakeholder analysis for policy engagement

Theme	Terms used	Operational definition
**Influence, importance and agreement analysis of stakeholders**	Level of influence	Stakeholders' influence will be determined according to each stakeholder group's perception and views on who is important in terms of urban health care delivery.
	Level of agreement	Stakeholders' agreement will be determined according to how much each stakeholder agreed.
	Level of importance	The stakeholders' importance will be determined according to how important each stakeholder group is to the other groups.
**Power and leadership**	Overall power	Power of a stakeholder-group will be assessed as compared to all other groups in Bangladeshi urban healthcare delivery system. Power of stakeholders will be measured as the product of multiplication of influence and importance.
	Relative position	Relative position of each stakeholder group will be assessed by comparing one group's position to other groups in broader scenario.
**Relative positions of**	Drivers	Stakeholders who have high level of importance as well as high level of influence on public sector health care delivery system

For peer review only - http://bmjopen.bmj.com/site/about/guidelines.xhtml

**stakeholders**	Supporters	Stakeholders who have high level of importance but low level of influence on urban healthcare delivery system
	Bystanders	Stakeholders who have low level of importance and low level of influence on urban healthcare delivery system
	Abstainers	Stakeholders who have no influence and no importance on urban healthcare delivery system
	Blockers	Stakeholders who have low level of importance but high level of influence on urban healthcare care delivery system.

For the *policy engagement analysis,* KIIs with stakeholders will be examined to understand interactions between actors, content, context and processes with respect to ICT policy uptake, with a view to identifying entry points for policy advocacy and the sustainable incorporation of ICT in health systems. Interviews will be analyzed using a priori codes drawn from the Policy Engagement Framework. Of additional interest in this analysis is understanding the mechanisms and processes of health policy development and how other policies may be important in efforts to integrate ICT into health systems. Based on these insights, a Health Policy for ICT process flowchart will be prepared. According to the policy engagement framework we will also seek information on how context is considered and dealt with when policies are formed and what processes need to be changed to more effectively integrate UHA into the system.[22]

For peer review only - http://bmjopen.bmj.com/site/about/guidelines.xhtml

For *quantitative data*, simple descriptive analysis will be performed to show user rates over time. The set of parameters to be analyzed are: number of users who accessed UHA, type of user, scope of UHA use, and types of problems faced. For data analysis, software like MS Excel and STATA will be used as appropriate.

The *cost* of implementing UHA will be estimated using a direct approach. Average cost for each activity will be calculated. All supply-side inputs will be identified, quantified, and valued through record review and KIIs. Shared cost items, including salary, buildings, furniture, supervision, transportation, and vehicles will be identified through observation and interviews of relevant personnel. By considering the nature of inputs, these will be categorized into capital, recurrent as well as fixed and variable cost items. Shared costs will be apportioned by proportion of the time-involvement of the relevant items (office rent, common vehicle) to different activities. Inputs will be identified using discussion with relevant personnel, observation, and record review. If unavailable, market prices will be applied to estimate costs. The capital items will be annualized, and common costs will be apportioned as per requirement. Annual values of capital items will be estimated from their expected useful life years and annuitization will be done using 3% discounting rate whenever applicable.[34] The study will allocate the cost for shared items (e.g. office space, appliances) by using actual utilization of items for this activity. Utilization information for shared items will be collected from responsible project staff.

Finally, the cost for shared items will be estimated by multiplying percentage-use information with the total cost of the items. Total cost will be calculated by summing up the start-up and

For peer review only - http://bmjopen.bmj.com/site/about/guidelines.xhtml

implementation costs. Relative contribution of start-up and implementation cost will be calculated. The cost-drivers in each activity will be identified considering the larger share of total cost.

Process documentation

In addition to all these activities, the study investigators will systematically document implementation processes for policy uptake and institutionalization of UHA, focusing particularly on contextual factors and their influence on implementation using a process documentation template. Process documentation of this "pilot phase" of UHA institutionalization will generate supporting knowledge to be applied the phases of scale-up and sustain, as specified in the WHO/PATH toolkit.

Patient and public involvement

There will be no direct patient or public involvement in this implementation research. However, a Technical Advisory Group (TAG) will be formulated for project governance, which will consist of representatives from Government, development partners, NGOs, academicians and senior researchers and urban health actors. Regular meetings will be held with partners and staff for problem solving. At the end of the study, the TAG will comment on the study findings and contribute to the dissemination plan.

Ethics and Dissemination

The institutional review board of icddr,b is comprised of two committees: the Research Review Committee (RRC) and Ethical Review Committee (ERC). This study has received approval from

For peer review only - http://bmjopen.bmj.com/site/about/guidelines.xhtml

both of the committees, which provided a thorough and critical review of the protocol's technical and ethical aspects. Participants will be asked for written consent prior interviewing and will remain anonymous and unidentifiable. Tape recorders will be used to record discussions bur only after obtaining consent. All other form of data will be kept in locked storage, or controlled access folders, allowing only investigators of the study and members of the ERC of icddr,b to access information, if needed.

Findings from this research will be disseminated at various levels to develop interest and support from a wide variety of audiences i.e. public, private, NGO, civil society, and donors. In doing so, we hope to build a diverse constituency of individuals and organizations willing and able to translate evidence yielded by the study, into policy action.

Local dissemination: Findings will be presented to relevant local administrators, development partners and NGOs and other relevant parties (local health practitioners), researchers.

National dissemination: A series of interactive workshops and briefing sessions with various stakeholders will be arranged to create linkages with national fora. The main aim will be to translate findings in a more visual and engaging formats, i.e. research briefs and interactive project brochures, to reach a range of stakeholders.

International dissemination: This will include publishing findings in peer-reviewed journals and presenting in scientific forums, conferences, and symposiums, and linking with international learning platforms. The main objective is to contribute to global knowledge about context

For peer review only - http://bmjopen.bmj.com/site/about/guidelines.xhtml

specific strategies to incorporate ICTs into health systems, and challenges that must be anticipated for policy uptake necessary to introduce, scale up and sustain MNCH related ICTs in similar LMICs.

For peer review only

For peer review only - http://bmjopen.bmj.com/site/about/guidelines.xhtml

REFERENCES

1. Adams AM, Ahmed SM, Evans TG. Universal health care in Bangladesh–promises and perils. *Lancet Glob Health* 2018;6:e10-11.
2. Ahmed SM, Islam FK, Bhuiya A, ed. *Bangladesh Health Watch Report 2014: Urban Health Scenario: Looking Beyond 2015.* Dhaka: James P Grant School of Public Health, BRAC University, 2015.
3. Chowdhury AMR, Bhuiya A, Chowdhury ME, *et al*. The Bangladesh paradox: exceptional health achievement despite economic poverty. *Lancet* 2013;382:1734-45.
4. National Institute of Population Research and Training. *Bangladesh Urban Health Survey 2013; Final Report*. Dhaka: 4. National Institute of Population Research and Training, 2013.
5. National Institute of Population Research and Training. *Bangladesh Demographic and Health Survey 2014: Key Indicators*. Dhaka: 4. National Institute of Population Research and Training, 2016.
6. United Nations. Department of Economic and Social Affairs Population Dynamics.. World Urbanization Prospects2018. Available: https://esa.un.org/unpd/wup/publications/Files/WUP2018-KeyFacts.pdf
7. Alam SMN. Health service delivery: the state of government-non-government relations in Bangladesh. *Public Adm Dev* 2011;31:273-81.
8. Afsana K, Wahid SS. Health care for poor people in the urban slums of Bangladesh. *Lancet* 2013;382:2049-51.
9. Huque R, Barkat A, Sabina N. Public health expenditure: equity, efficacy and universal health coverage. *In*: Ahmed SM, Mahmud S, Evans TG, *et al*. ed. *Bangladesh Health*

For peer review only - http://bmjopen.bmj.com/site/about/guidelines.xhtml

Watch Report 2011: Moving towards Universal Health Coverage. Dhaka: James P Grant School of Public Health, BRAC University, 2012:25-32.

10. Osman F. Public health, urban governance and the poor in Bangladesh: policy and practice. *Asia-Pacific Dev J* 2009;16:27-58.

11. Matthews Z, Channon A, Neal S, Osrin D, Madise N, Stones W. Examining the "urban advantage" in maternal health care in developing countries. *PLoS Med* 2010;7:e1000327.

12. Local Government Division, Government of the People's Republic of Bangladesh. Urban Primary Health Care Services Delivery Project. Available: https://www.adb.org/projects/42177-024/main

13. Nutley T, Reynolds HW. Improving the Use of Health Data for Health System Strengthening. *Glob Health Action* 2013;6.

14. Few S. Data Visualization for Human Perception. In: *The Encyclopedia of Human – Computer Interaction*, 2nd Ed. Available: https://www.interaction-design.org/literature/book/the-encyclopedia-of-human-computer-interaction-2nd-ed/data-visualization-for-human-perception

15. World Health Organization. *Creating a Master Health Facility List*. Geneva: World Health Organization, 2013.

16. Rose-Wood A, Heard N, Thermidor R, *et al*. Development and use of a master health facility list: Haiti's experience during the 2010 earthquake response. *Glob Health Sci Pract* 2014;2:357-65.

17. World Health Organization. *Toolkit on monitoring health systems strengthening Measuring Health Systems Strengthening and Trends: A Toolkit for Countries*. Geneva: World Health Organization, 2008. Available at:

For peer review only - http://bmjopen.bmj.com/site/about/guidelines.xhtml

https://www.who.int/healthinfo/statistics/toolkit_hss/EN_PDF_Toolkit_HSS_Introduction.pdf

18. World Health Organization/PATH. *Planning an Information Systems Project: A Toolkit for Public Health Managers*. Seattle: PATH 2013.
19. World Health Organization. Master facility list resource package: guidance for countries wanting to strengthen their master facility list. Available at: https://www.who.int/healthinfo/country_monitoring_evaluation/mfl/en/
20. Adams AM, Ahmed S., Hasan SM,. *et al*. *Mapping the Urban Healthcare Landscape in 5 City Corporations, Bangladesh*. Dhaka: icddr,b, 2015.
21. icddrb. The Urban Health Atlas. Available: http://www.urbanhealthatlas.com
22. Buse K, Booth D, Murindwa G, *et al*. *Donors and the Political Dimensions of Health Sector Reform: The Cases of Tanzania and Uganda*. London: Overseas Development Institute, 2008.
23. Peters DH, Adam T, Alonge O, Agyepong IA, Tran N. Implementation research: what it is and how to do it. *BMJ* 2013;347:f6753.
24. Archangel N. *The critical issues affecting the introduction of Health Management Information Systems in developing countries in Africa*. Amsterdam: Universiteit van Amsterdam, 2007.
25. Kilbourne AM, Neumann MS, Pincus HA, Bauer MS, Stall R. Implementing evidence-based interventions in health care: application of the replicating effective programs framework. *Implement Sci.* 2007;2:42.
26. Guest G, Bunce A, Johnson L. How many interviews are enough? An experiment with data saturation and variability. *Field Methods.* 2006;18:59-82.

For peer review only

27. Crouch M, McKenzie H. The logic of small samples in interview-based qualitative research. *Soc Sci Inf.* 2006;45:483-99.

28. Bryson JM. What to do when stakeholders matter: stakeholder identification and analysis techniques. *Public Manag Rev*. 2004;6:21-53.

29. Bryson JM. *Strategic planning for public and nonprofit organizations: A guide to strengthening and sustaining organizational achievementVol 1.* San Francisco, CA: Jossey-Bass Publishers, 1995.

30. Moore MH. *Creating public value: Strategic management in government*. Cambridge, MA: Harvard University Press, 1995.

31. Gold MR, Siegel JE, Russell LB, *et al*. ed. *Cost-effectiveness in Health and Medicine 1ed*. New York: Oxford University Press, 1996.

32. Drummond MF, Sculpher MJ, Claxton K, *et al*. *Methods for the economic evaluation of health care programmes*. Oxford: Oxford University Press 2015.

33. World Health Organization. *Comprehensive Multi-Year Planning (cMYP): A Tool and User Guide for cMYP Costing and Financing*. Geneva:World Health Organization, 2014.

34. Fiedler JL, Villalobos CA, De Mattos AC. An activity-based cost analysis of the Honduras community-based, integrated child care (AIN-C) programme. *Health Policy Plan* 2008;23:408-27.

For peer review only - http://bmjopen.bmj.com/site/about/guidelines.xhtml

Figure 1. Modified conceptual framework for Information Communication and Technology (ICT) implementation in developing countries

For peer review only

For peer review only - http://bmjopen.bmj.com/site/about/guidelines.xhtml

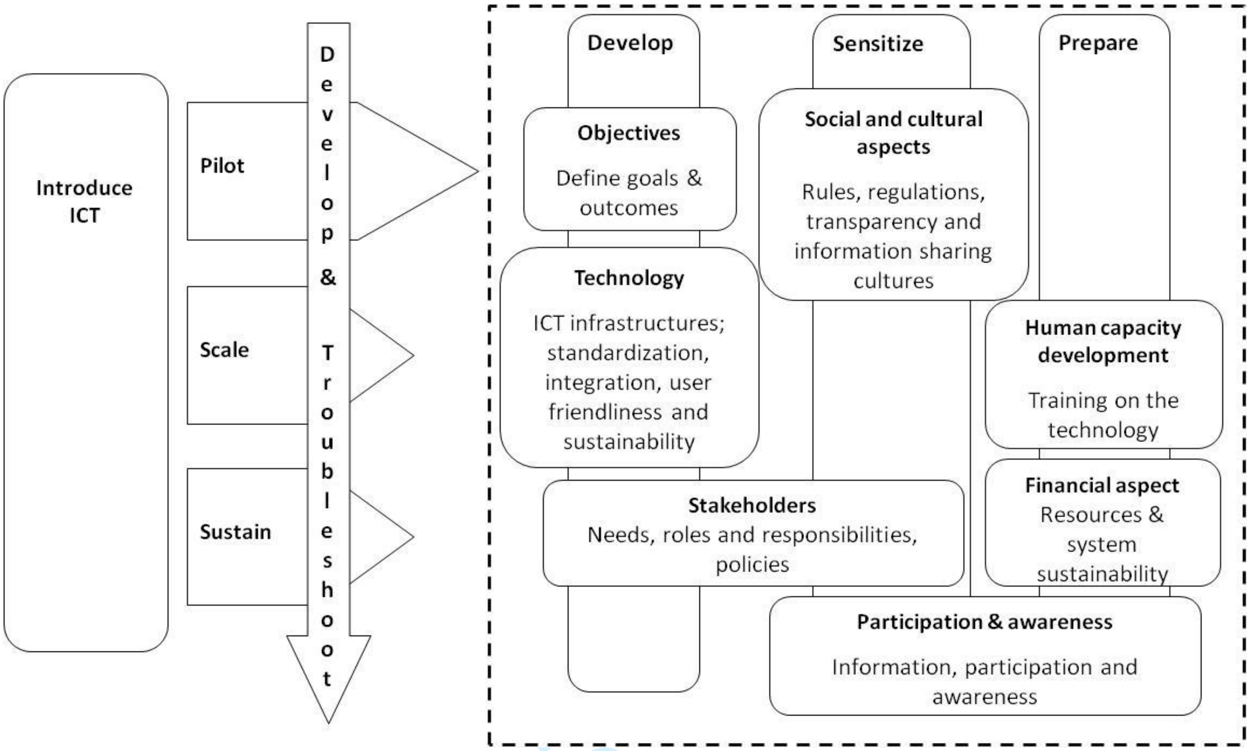

Figure 1. Modified Conceptual framework for Information Communication and Technology (ICT) implementation in developing countries

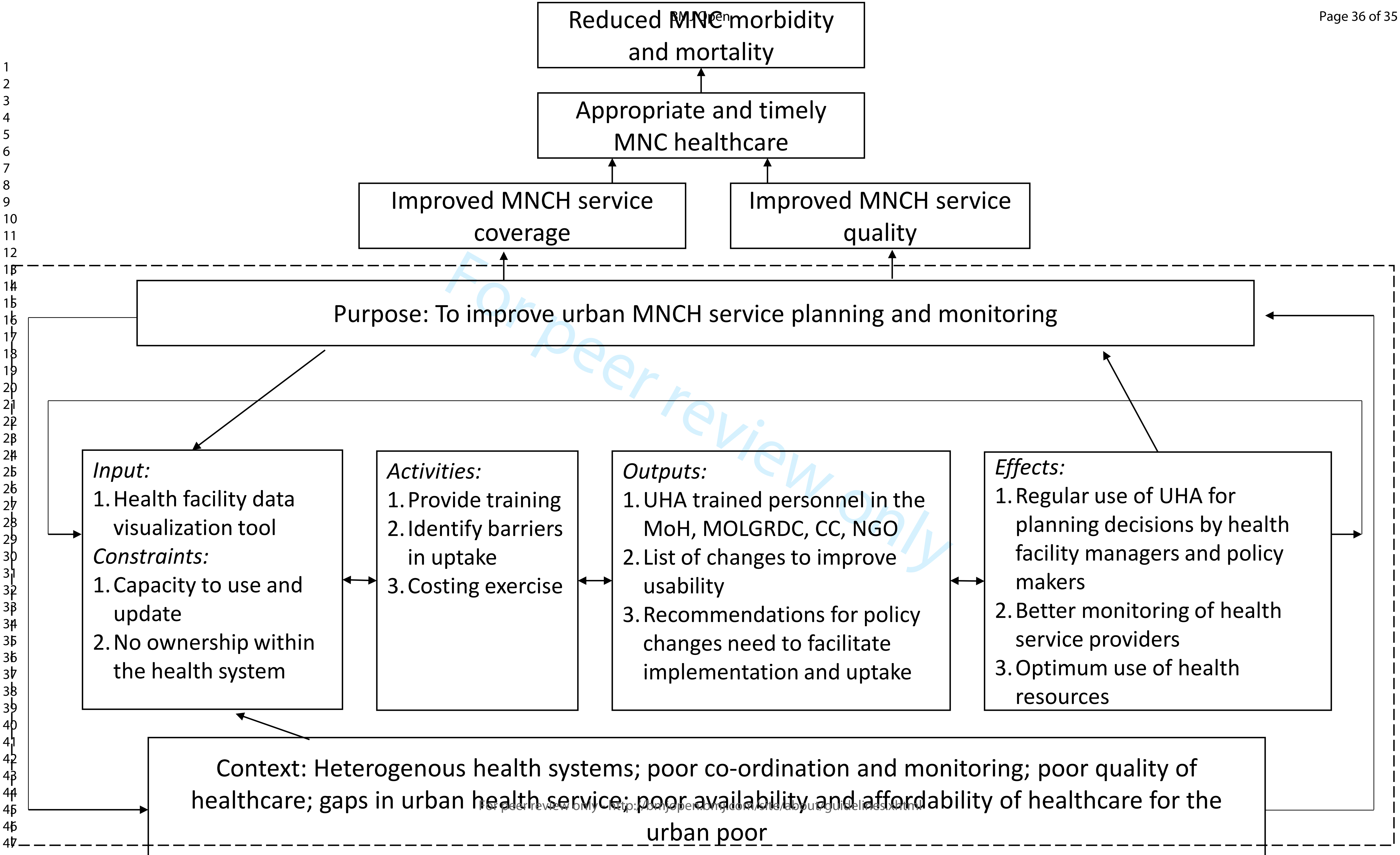

For peer review only

Making Information and Communications Technologies (ICTs) work for health: protocol for a mixed-methods study exploring processes for institutionalizing geo-referenced health information systems to strengthen Maternal Neonatal and Child Health (MNCH) service planning, referral and oversight in urban Bangladesh

Journal:	*BMJ Open*
Manuscript ID	bmjopen-2019-032820.R3
Article Type:	Protocol
Date Submitted by the Author:	24-Sep-2020
Complete List of Authors:	Islam, Rubana ; University of New South Wales, School of Public Health and Community Medicine<br>Adams, Alayne Mary ; McGill University,<br>Hasan, Shaikh Mehdi ; International Centre for Diarrhoeal Disease Research Bangladesh<br>AHMED, RUSHDIA; International Centre for Diarrhoeal Disease Research Bangladesh, Health Systems and Population Studies Division<br>Bhattacharyya, Dipika Shankar; International Centre for Diarrhoeal Disease Research, Health Systems and Population Studies Division<br>Shafique, Sohana; International Centre for Diarrhoeal Disease Research, Health Systems and Population Studies Division
**Primary Subject Heading**:	Public health
Secondary Subject Heading:	Global health, Health policy, Health informatics, Health services research
Keywords:	Urban health, maternal, neonatal and child health, information communication and technology, geographic information systems, health management information systems, Bangladesh

I, the Submitting Author has the right to grant and does grant on behalf of all authors of the Work (as defined in the below author licence), an exclusive licence and/or a non-exclusive licence for contributions from authors who are: i) UK Crown employees; ii) where BMJ has agreed a CC-BY licence shall apply, and/or iii) in accordance with the terms applicable for US Federal Government officers or employees acting as part of their official duties; on a worldwide, perpetual, irrevocable, royalty-free basis to BMJ Publishing Group Ltd ("BMJ") its licensees and where the relevant Journal is co-owned by BMJ to the co-owners of the Journal, to publish the Work in this journal and any other BMJ products and to exploit all rights, as set out in our licence.

The Submitting Author accepts and understands that any supply made under these terms is made by BMJ to the Submitting Author unless you are acting as an employee on behalf of your employer or a postgraduate student of an affiliated institution which is paying any applicable article publishing charge ("APC") for Open Access articles. Where the Submitting Author wishes to make the Work available on an Open Access basis (and intends to pay the relevant APC), the terms of reuse of such Open Access shall be governed by a Creative Commons licence – details of these licences and which Creative Commons licence will apply to this Work are set out in our licence referred to above.

Other than as permitted in any relevant BMJ Author's Self Archiving Policies, I confirm this Work has not been accepted for publication elsewhere, is not being considered for publication elsewhere and does not duplicate material already published. I confirm all authors consent to publication of this Work and authorise the granting of this licence.

For peer review only

Title: Making Information and Communications Technologies (ICTs) work for health: protocol for a mixed-methods study exploring processes for institutionalizing geo-referenced health information systems to strengthen Maternal Neonatal and Child Health (MNCH) service planning, referral and oversight in urban Bangladesh

Authors

Rubana Islam (rubana.islam@student.unsw.edu.au)[1*]

Alayne Adams (alayne.adams@gmail.com)[2*]

Shaikh Mehdi Hasan (smehdi@icddrb.org)[3]

Rushdia Ahmed (ahmed.rushdia@yahoo.com)[3]

Dipika Shankar Bhattacharyya (dipikashankar@gmail.com)[3]

Sohana Shafique (sohana.shafique@icddrb.org)[3**]

Author Affiliations

[1] School of Public Health and Community Medicine, University of New South Wales, Sydney, Australia

[2] Department of Family Medicine, Faculty of Medicine and Health Sciences, McGill University, Montréal, Québec, Canada

[3] Universal Health Coverage Programme, Health Systems and Population Studies Division, icddr,b, Dhaka, Bangladesh

*These authors contributed equally for this work.

For peer review only - http://bmjopen.bmj.com/site/about/guidelines.xhtml

**Corresponding author/request for reprints:

Dr. Sohana Shafique

Assistant Scientist and Deputy Project Coordinator

Universal Health Coverage programme

Health Systems and Population Studies Division

icddr,b, Mohakhali, Dhaka. Bangladesh.

Email: sohana.shafique@icddrb.org

Phone: +880-2-9827001-10, ext. 2273

Word Count: 4550

Author Contributions

RI & AA conceptualized the study. RI & AA prepared the first draft of the manuscript. SMH, RA, DSB and SS revised the manuscript. RI & AA reviewed critically for important intellectual content; SS revised the version submitted with inputs from all other co-authors.

Acknowledgements

This research study was funded by International Development Research Centre (IDRC). icddr,b acknowledges with gratitude the commitment of IDRC to its research efforts. icddr,b is also grateful to the Governments of Bangladesh, Canada, Sweden and the UK for providing core/unrestricted support.

For peer review only - http://bmjopen.bmj.com/site/about/guidelines.xhtml

Competing interests

The authors declare no competing interests.

Funding

This work was supported by International Development Research Centre (IDRC), Canada (Grant Number: 108218-001).

Study period

2016-2019

Data statement section

Data will be handled according to the principles of the icddr,b policies and guidelines of the International Development Research Centre, Canada. Since this is a protocol paper, no data are available.

For peer review only

Abbreviations

ADB	Asian Development Bank
CCs	City Corporations
DGHS	Directorate General of Health Services
ERC	Ethical Review Committee
GIS	Geographic Information System
HMIS	Health Management Information System
ICT	Information Communication and Technology
IDI	In-depth Interviews
KII	Key Informant Interviews
LMICs	Low and Middle Income Countries
MNCH	Maternal, newborn and child health
MOHFW	Ministry of Health and Family Welfare
MOLGRDC	Ministry of Local Government, Rural Development and Cooperatives
NGOs	Non-Government Organizations
PHC	Primary Health Care
RRC	Research Review Committee
SDGs	Sustainable Development Goals
UHA	Urban Health Atlas
UHC	Universal Health Coverage
WHO	World Health Organization

For peer review only

For peer review only - http://bmjopen.bmj.com/site/about/guidelines.xhtml

ABSTRACT

Introduction: Disparities in health outcomes and access to maternal neonatal and child health (MNCH) are apparent among urban poor compared to national, rural or urban averages. A fundamental first step in addressing inequities in MNCH services is, knowing what services exist in urban areas, where these are located, who provides them, and who uses them. This study aims to institutionalize the Urban Health Atlas (UHA) - a novel ICT tool - to strengthen health service delivery and oversight and generate critical evidence to inform health policy and planning in urban Bangladesh.

Methods and analysis: This mixed-method implementation research will be conducted in four purposively selected urban sites representing larger and smaller cities. Research activities will include an assessment of information needs and task review analysis of information users, stakeholder mapping, and cost estimation. To document stakeholder perceptions and experiences, Key Informant Interviews (KIIs) and In-depth Interviews (IDIs) will be conducted along with desk reviews to understand MNCH planning and referral decisions. The UHA will be refined to increase responsiveness to user needs and capacities, and hands-on training will be provided to health managers. Cost estimation will be conducted to assess the financial implications of UHA uptake and scale-up. Systematic documentation of the implementation process will be undertaken. Policy decision-making and ICT health policy process flowcharts will be prepared using desk reviews and qualitative interviews. Thematic analysis of qualitative data will involve both emergent and a priori coding guided by WHO PATH toolkit and Policy Engagement Framework. Stakeholder analysis will apply standard techniques and measurement scales. Descriptive analysis of quantitative data and cost estimation analysis will also be performed.

For peer review only - http://bmjopen.bmj.com/site/about/guidelines.xhtml

Ethics and dissemination: The study has been approved by the Institutional Review Board of icddr,b (# PR–16057). Study findings will be disseminated through national and international workshops, conferences, policy briefs and peer-reviewed publications.

Keywords

Urban health, maternal, neonatal and child health, information communication and technology, geographic information systems, health management information systems, Bangladesh.

Article Summary

Strengths and limitations of this study

- This mixed method implementation research is among the first in Bangladesh to explore processes for institutionalizing geo-referenced health information systems to strengthen MNCH service planning, referral, and oversight in urban areas.
- A conceptual framework specific to ICT tools and their implementation has been developed to guide the research.
- Implementation partnership with the Government will be established to ensure post-implementation maintenance of the geo-referenced health information system.
- Acknowledging the potential contribution of patients and civil society groups to ICT uptake, these groups are not engaged in the health planning exercise for logistic reasons.

For peer review only

For peer review only - http://bmjopen.bmj.com/site/about/guidelines.xhtml

INTRODUCTION

Bangladesh has embraced the Sustainable Development Goal-3 (SDGs) of achieving universal health coverage by 2030,[1] however, challenges related to rapid population growth, pluralistic health systems, and lack of governance, among others, are substantial.[2] Although Bangladesh has made extraordinary progress in reducing maternal and child mortality,[3] there are significant disparities in health-related outcomes and access to maternal neonatal and child health (MNCH) services stratified along both socioeconomic and geographic dimensions. Health indicators are far worse in urban slums than both non-slum urban areas and the national average.[4] Nationally, the mortality rate for children under five years of age is 65 per 1,000 live births and 49 per 1,000 live births in rural areas while the rate is 81 per 1000 live births among urban slum residents.[4, 5] Undergoing rapid urbanization, the country is projected to become over 50% urban by 2040, with almost one-third of urban residents living in slums.[6] Persistent inequities in key MNCH indicators in urban areas highlight the need to focus on issues of service coverage, access, quality and timely and appropriate referral as urgent policy priorities.

The Bangladesh urban health system is a smorgasbord of service providers characterized by inadequate coordination and regulation, and geographic and socioeconomic inequities in healthcare access.[7, 8] Several reasons have been proposed for inefficiencies in the system including poor planning and management capacity, weak coordination among the authorities, lack of clear, separate roles and responsibilities for the various authorities, service coverage gaps, and human resource management issues.[9, 10] Of particular concern in urban areas is the lack of adequate public primary care infrastructure and services which disproportionately impacts the urban poor, and poses significant challenges to the country's aspirations to meet the goal of Universal Health Coverage by 2030.[11] One consequence of limited formal primary healthcare

For peer review only - http://bmjopen.bmj.com/site/about/guidelines.xhtml

services in urban areas[12] is the emergence of the private sector in health including the proliferation of informal providers such as pharmacies on which many of the urban poor rely. The formal private sector is equally massive, accounting for 80% of over 3500 hospitals in Bangladesh, and an even greater percentage in urban areas. Lack of regulation of this sector has resulted in concerns about quality of care and financial accessibility, especially for the urban poor.[10]

A fundamental first step in addressing inequities in urban healthcare access is an in-depth understanding of what services exist, their location, who provides them, and who utilizes them. A strong health management information system (HMIS), an essential component of sound programme development and implementation. Enabling the use of data for strategic decision-making, better governance, institutionalized HMIS systems represent the foundation upon which improvements in health outcomes can be monitored and greater accountability ensured.[13, 14] A Master Facility List (MFL) is a crucial constituent of HIS and permits the linkage of sub-systems within national HIS architecture.[15,16] MFL is advocated by The World Health Organization (WHO) as an effective means of ensuring better governance including systematic reporting and monitoring supervision.[17,18] MFLs like UHA are expected to facilitate health service planning and management through mapping or visualizing the distribution of health services and resources. It can also assist health service providers in identifying appropriate referral facilities for patients.[19] These functions can help improve equitable service coverage and reduce delays in receiving appropriate care, which in turn can impact health outcomes such as maternal and child mortality among the urban poor. A theory of change is provided in supplemental file 1.

For peer review only

For peer review only - http://bmjopen.bmj.com/site/about/guidelines.xhtml

Realizing the critical role that health information systems play in health management and building on political commitment towards "Digital Bangladesh", the Bangladesh Directorate General of Health Services (DGHS), Ministry of Health and Family Welfare (MOHFW) is implementing the District Health Information Software-2 (DHIS2) with support from development partners. While the system has been rolled out nationally, information is largely confined to public healthcare facilities. In urban areas, with the exception of large public hospitals and a number of NGOs involved in primary care provision, data are particularly sparse, especially for the massive private sector.

Urban Health Atlas: A novel ICT tool

Addressing this information gap, icddr,b has created a geo-referenced heath facility database for nine major cities and municipalities across Bangladesh. This dataset consists of a census of all healthcare facilities and the services they provide along with their geo locations.[20] To enable the practical application of this dataset, an Information Communication and Technology (ICT) tool called *Urban Health Atlas* (UHA) was developed (http://urbanhealthatlas.com).[21] This GIS-based interactive online tool displays health facility data visually and permits their manipulation for better healthcare planning and decision making. Providing detailed information on the location and services available at public and private health facilities, it allows users to examine gaps and duplication in service provision, assess the coverage of emergency services and the availability of doctors in a 24-hour period, calculate the shortest distance to referral facilities from any location, and determine whether a given facility is licensed and registered. This information is particularly useful in helping healthcare planners and policy makers make informed decisions around the distribution and monitoring of healthcare facilities and services,

For peer review only - http://bmjopen.bmj.com/site/about/guidelines.xhtml

and health human resources. For the general public, the tool holds promise in locating a desired healthcare service that is closest in distance and indicating the shortest path to get there.

A key strength of this dataset is its inclusion of private-for-profit healthcare facilities, from pharmacies to hospitals, in addition to public and private not-for-profit healthcare provision. The UHA prototype has been demonstrated both nationally and internationally, and generated a great deal of interest and useful feedback. In Bangladesh, its promise has intrigued multiple stakeholder groups, ranging from the Directorate General of Health Services (DGHS) responsible for national healthcare planning, local government officials, private not-for-profit or non-government organizations (NGOs), service providers and development partners. In the context of significant investments in urban health systems strengthening that are in pipeline, and absence of urban data in the country's national health information system (DHIS2), UHA is widely regarded as timely and useful in the context of current urban health planning processes, and many discussions about its formal linkage to and institutionalization within existing health information systems have occurred.

However, due to the complexity of these kind of data, they risk being underutilized for health policy and planning unless specific efforts are attempted to make them more accessible to non-technical, policy and other local level stakeholders.[22] In the context of UHA, these efforts have included making the data available on the DGHS webpage, and organizing dissemination events in city corporations and municipalities. However, beyond anecdotal reports, there is no systematic information on whether the tool is being used by stakeholders, and how it could be improved to better meet their needs. The purpose of this study, therefore, is to pilot and refine the UHA for use in service delivery planning and referral, and by generating evidence on its utility,

For peer review only - http://bmjopen.bmj.com/site/about/guidelines.xhtml

For peer review only

inform and strengthen advocacy for and action around its institutionalization into the government system. A focus on MNCH service delivery was chosen to circumscribe the development of training materials, and to clearly delimit the range of stakeholders that should be engaged.

Study Aims

Three specific aims are identified in seeking to institutionalize the UHA for MNCH service delivery, planning, and referral into the government system:

1. To document stakeholder perceptions and experiences in adopting a tool that enables use of health facility information for strategic planning, day-to-day decision-making, control and oversight, and improved administrative efficiency of urban MNCH services.
2. To identify policy and programmatic entry points that will facilitate broader use of geo-referenced health facility information and its regular update.
3. To estimate costs associated with bringing geo-referenced facility listing into the government system

METHODS

This Implementation Research (IR) focuses on the factors and processes that influence uptake, use and scale-up of ICT tools like UHA. The study will explore barriers in usability, understandability, and utility, as well as policy and other requirements needed to support its systematic implementation in the real world setting of healthcare planning, referral, and oversight. The primary audiences of this research are managers and decision makers in the urban healthcare sector of Bangladesh.

Study design and participants

For peer review only - http://bmjopen.bmj.com/site/about/guidelines.xhtml

The proposed implementation research employs a mixed method research approach. Mixed-method research is a widely used approach in IR.[23] We will assess the uptake of UHA by the MNCH-related planners and decision makers over a 3-year period from 2016-2019. The specific IR variables to be assessed are adoption, appropriateness, feasibility, and implementation cost [23]. Many IR frameworks exist, however it is advised to use a framework befitting program parameters [23]. For this reason, we identified a toolkit specific to the development and use of ICT tools and formulated our conceptual framework accordingly. This helped to operationalize the research as an ICT intervention versus a clinical or health service level intervention, while still retaining some of the features of common IR frameworks, including concerns with guided implementation and innovation, sustainability, and stakeholder input.[23]

The WHO PATH toolkit was published by WHO and PATH to guide the introduction and implementation of information and communications technology (ICT) in health information systems.[18] The introduction of a new ICT tool is commonly accompanied by challenges that must be overcome. Before scaling-up, therefore, it is important to conduct rigorous product planning and feasibility testing, and to identify and engage key stakeholders. The toolkit identifies three main phases of an ICT project: pilot, scale, and sustain. In the pilot phase, the phase addressed in this study, a solution (UHA) is developed based on program needs and priorities, and tested on a small-scale to measure outcomes, impacts, and costs, and identify potential improvements. Several other factors influence the introduction of the HMIS in developing countries including planning, stakeholder roles and responsibilities, cultural aspects, human capacity, financial aspects sustainability etc. (see figure 1).[24] The elements of this ICT-informed framework are similar to that of the different phases of the “replicating effective programs framework” used in IR.[25] For instance, stakeholder needs in our framework are

For peer review only - http://bmjopen.bmj.com/site/about/guidelines.xhtml

addressed under the identifying implementation barriers step (pre-conditions phase), the orientation step of the pre-implementation phase is similar to participation and awareness and financial aspect elements in our framework, and training and technical assistance of the implementation phases are addressed through the human capacity development component.

In order to explore user perceptions, and policy and programmatic entry points, the policy engagement framework [22] also will be employed to prospectively analyze policy that incorporates strategies for change. This framework will confer a systematic approach to the ongoing collection, analysis and use of political information (e.g. concerning actors, their interests, institutions, ideas, and policy processes and context) that can alter the balance of power between those in support of and those resisting change by enabling pro-reformers to intervene more effectively in the policy process.[22]

Study sites

Two city corporations (CCs) – namely Dhaka North City Corporation (DNCC) and Dhaka South City Corporation (DSCC) – and two municipalities, Jessore and Dinajpur, which have geo-referenced health facility data available from previous mapping exercise of icddr,b[21] will be purposively selected. As smaller cities, Jessore and Dinajpur municipalities present marked differences from CCs in terms of size, structure, capacity, and challenges posed.

Sample size

Through the stakeholder mapping, key urban MNCH decision-making actors at both national and local levels will be engaged to help identify potential users for UHA. This group will constitute

For peer review only - http://bmjopen.bmj.com/site/about/guidelines.xhtml

our study respondents and training participants. When determining sample size for qualitative research, Guest et al. propose that a homogenous group of respondents 12 interviews is sufficient for reaching data saturation.[26] It is also asserted that a sample under 20 respondents allows qualitative researchers to establish and maintain effective relationships with study participants, and thus enhances the validity of the research.[27] For these reasons, we will sample 15-16 respondents for each of our activities. The sampling strategy, type, and number of respondents for each study activity are provided in table 1.

Table 1 Sampling strategy and sample type for each activity of the study

Activity and focus	Data collection methods	Sampling strategy	Respondent group	Sample size
**Task Review** (document how MNCH planning and referral decisions are currently made)	KIIs	Opportunistic/ Emergent sampling; Snowball sampling	–Urban health systems actors<br>–National and local government officials<br>–NGO programme managers	16
	Desk review	N/A	N/A	N/A

For peer review only

**User Need and User Experience** (explore user preferences, task needs and experiences)	IDIs (on user needs)	Opportunistic/ Emergent sampling; Snowball sampling	–Policy makers within the MOHFW<br>–Members of the Urban Health Cell of MOLGRDC<br>–Managers at City Corporations<br>–NGO programme managers	16
**Policy Engagement** (understand interactions between content, context, actors and processes of policy advocacy and entry points for sustainable	KIIs	Opportunistic/ emergent sampling; Snowball sampling	–Urban health systems actors<br>–National and local government officials<br>–NGO programme managers	15
incorporation of ICT into health systems)	Desk review (urban health and ICT related policies)	N/A	N/A	N/A

For peer review only - http://bmjopen.bmj.com/site/about/guidelines.xhtml

ICT, Information Communication and Technology; IDI, In-depth Interviews; KII, Key Informant Interviews; MNCH, Maternal, newborn and child health; MOHFW, Ministry of Health and Family Welfare; MOLGRDC, Ministry of Local Government, Rural Development and Cooperatives.

Implementation procedure of the pilot

The study implementation is envisaged to begin with stakeholder sensitization and partnership-building with government. This is critical because the research will work closely with the government health system in urban areas. In addition, an intervention will be conducted consisting of capacity building sessions around the use of the UHA for government and other urban health planners and managers. Details of these activities are provided below.

Stakeholder consultation and engagement: Two stakeholder consultation workshops will be carried out to identify and engage key stakeholders to create research buy-in and to begin the process of UHA advocacy. Detailed information on identification and mapping of stakeholders has been described in the data collection section. Ideas will be generated for uptake, regular use, and update of UHA.

Partnership development with Government: Implementation partnerships with the Management Information System (MIS) of the DGHS, MOHFW will be developed and a Memorandum of Understanding will be signed between icddr,b and DGHS. Permission letters will also be obtained from mayors of CCs and municipalities.

For peer review only - http://bmjopen.bmj.com/site/about/guidelines.xhtml

Development of training materials: A training manual on Urban Health Atlas (UHA) will be prepared to guide UHA capacity building workshops including case studies, guidelines for group work and hand-on activities, pre-test/post-test questionnaires, etc.

UHA workshops: In each study site, a 2-day UHA workshop, and subsequent 1-day refresher course, will be organized with a selected group of health workers and managers drawn from local government, and NGOs. Institutional agreements and permissions will be sought in advance from local government institutions and the health ministry as appropriate. Training sessions will provide an introduction to current urban health challenges, followed by an overview and demonstration of the Urban Health Atlas and its functions. Hands on training, group work and case studies will be undertaken to familiarize users with UHA and to get their feedback on how it might be improved to better meet their needs.

Data collection

Interviews will be conducted by an experienced group of researchers trained in qualitative interviewing including a mixed method expert, two software programmers, one GIS expert and an economist. The team will begin data collection in Dhaka, then move to the municipalities. A period of rapport building with key stakeholders in each study site will be critical to the success of this research given known difficulties in accessing the Government sector. Utilizing existing networks and negotiation skills will be especially important in opening doors and initiating discussion. The UHA tool will be assessed for impact on MNCH decision-making and outcomes. It is unlikely, however, that these effects would be apparent within three years of implementation. Thus, success of the tool will be determined based on user experiences as

For peer review only - http://bmjopen.bmj.com/site/about/guidelines.xhtml

specified by WHO PATH Toolkit i.e. better indicators for strategic planning, day-to-day decision-making, control and oversight, and reduced administrative burden.

Data collection methods for objective 1

To address objective 1, assessment of information needs and task review analysis will be done by desk review, in-depth interviews and click streams. Guidelines for qualitative interviews will be developed based on the WHO PATH toolkit's questions to measure success. Three qualitative research activities are envisaged to address this objective:

1. Key informant interviews (KIIs) with urban health system actors along with desk reviews to understand and document how MNCH planning and referral decisions are currently made.
2. In-depth interviews (IDIs) with potential UHA users to explore user preferences and task needs to refine the tool in advance of training.
3. IDIs with UHA users to understand their experiences and to document challenges and successes of using UHA for MNCH service decision-making during training and one- & three-months post-training.

In addition to qualitative assessments of user experiences, quantitative assessments of how different stakeholders are using data remotely will be made through (i) User's click streams; (ii) Task time devoted to different applications. Written feedback through online tools (i.e. Google Analytics) that facilitate remote testing will also be collected to generate more user-friendly functions that meet user needs.

For peer review only - http://bmjopen.bmj.com/site/about/guidelines.xhtml

Data collection methods for objective 2

To identify entry points that will facilitate broader use of geo-referenced facility information and its regular update, stakeholder mapping, policy mapping using desk review, KIIs, and stakeholder consultation workshop will be conducted. Guidelines for qualitative interviews will be developed using the policy engagement framework as a guide. Policy and programmatic entry points for the broader use and update of facility information, stakeholder analysis will be undertaken using Policy Engagement Framework as a guide. Stakeholders are identified as persons, groups, organization members or systems that affect or can be affected by a project/program/activity. Stakeholder analysis is an approach for generating knowledge about roles, behavior, inter-relation and intention of associated actors and their influence in implementation processes of a program or policy.[28] Given the importance of stakeholder satisfaction and support for the success of any program,[29,30] incorporating stakeholders' perspectives and needs is a critical step in gaining ownership around an ICT innovation like UHA and its incorporation into routine information systems, and use for decision-making. The following qualitative methods will be used to fulfill objective 2:

1. Stakeholder mapping including the identification and listing of stakeholder groups involved in urban health based on available literature and expert opinion.
2. A semi-structured guideline will be used to collect information during stakeholder consultation workshops to explore their respective interests, roles and responsibilities in urban health, their information needs, and perceptions of how they can contribute to institutionalizing UHA.

For peer review only - http://bmjopen.bmj.com/site/about/guidelines.xhtml

3. KIIs along with desk reviews will be undertaken to understand the processes of current health policy-making mechanisms and what other policies affect the integration of ICT in health.

Data collection methods for objective 3

To estimate cost of bringing facility listing into the government information system the total cost of ownership for UHA development and implementation will be estimated using an ingredient approach. Data will be collected through structured questionnaire, document review and KII. Cost will be estimated based on supply side aspects. The budget matrix will be developed with cost drivers proposed in the WHO PATH toolkit.

The cost for development and implementation of Urban Health Atlas (UHA) tools, coordination, and engagement of city corporations and DGHS will be estimated including both financial costs and economic costs of the program. Financial costs represent the actual expenditures on goods and services purchased. Economic costs include the estimated value of goods or services for which either there are no financial transactions or the price of a specific good did not reflect the cost of using it productively elsewhere.[31] The cost will be separated for start-up cost and implementation cost. The implementation cost comprises the costs required to run and maintain the ICT tools while executing intervention.[32]

All supply-side inputs will be identified, quantified, and valued through a facility-level inventory, record reviews and key informant interviews. Both fixed cost and variable cost will be captured. Shared cost items, including salary, buildings, furniture, supervision, transportation, and vehicles, will be identified through observation and interviews of relevant personnel. Shared

For peer review only - http://bmjopen.bmj.com/site/about/guidelines.xhtml

costs will be apportioned by proportion of the time-involvement of the relevant items (i.e. office rent, common vehicle) to different activities. The time of volunteers will be transformed into costs by using the minimum wage level of manual workers in Bangladesh. There are some examples of components included in ingredient approaches (table 2):[32,33]

Table 2 Components included in ingredient approaches for cost

Method Name	Methodology	Inputs	Activities
Ingredients approach	Quantities x price, personnel, percentage use	Personnel, ICT tools, vehicles	Personnel, ICT tools, vehicles

ICT, Information Communication and Technology.

To identify activities of the UHA tool implementation and their related inputs, a review of program documents and interviews with relevant personnel will be conducted. The unit price/salary information will be collected from responsible program management. In case of missing unit price of any items, the market price of those items will be collected. Semi-structured checklists for cost data collection will be developed considering the program context and using WHO PATH toolkit as a guiding framework. The budget matrix will be completed with help from key personnel associated with costing and budgeting identified during the stakeholder mapping and research team's own estimates.

Data analysis

For peer review only - http://bmjopen.bmj.com/site/about/guidelines.xhtml

A process flowchart for current decision-making practices will be prepared using the KII and Organizational process reviews. A list of user needs will be made and shared at a stakeholder consultation meeting to identify the most important and feasible functions to be added to UHA.

For *qualitative data*, an outline plan for data analysis will be prepared in advance of research along with a priori codes. These codes, mostly focusing on user experience, will be derived from the WHO PATH toolkit.[18] The analysis will be open to emerging themes as well. All interviews will be recorded provided consent has been obtained, but with simultaneous note taking in case of equipment failure. Data transcription will occur immediately following each interview, followed by translation. Data familiarization will involve reading transcripts repeatedly to surface emerging themes and identify any missed opportunities for further exploration. Transcripts will be coded using ATLAS-ti (version-7.5.7). A team approach to analysis will be employed to minimize individual biases. Inter-coder reliability will be checked. Group discussions of emerging themes and patterns in the data will be tested using data displays that allow more systematic pattern-testing across respondents.

For *stakeholder analysis*, stakeholders' influence, importance, and agreement will be explored applying standard techniques and measurement scales mentioned in table 3**.** A position diagram with level of agreement and level of influence will also be plotted to identify stakeholders who are already committed to work and help institutionalize UHA and those who need to be brought into agreement.

For peer review only - http://bmjopen.bmj.com/site/about/guidelines.xhtml

Table 3 Operational definitions for stakeholder analysis for policy engagement

Theme	Terms used	Operational definition
**Influence, importance and agreement analysis of stakeholders**	Level of influence	Stakeholders' influence will be determined according to each stakeholder group's perception and views on who is important in terms of urban health care delivery.
	Level of agreement	Stakeholders' agreement will be determined according to how much each stakeholder agreed.
	Level of importance	The stakeholders' importance will be determined according to how important each stakeholder group is to the other groups.
**Power and leadership**	Overall power	Power of a stakeholder-group will be assessed as compared to all other groups in Bangladeshi urban healthcare delivery system. Power of stakeholders will be measured as the product of multiplication of influence and importance.
	Relative position	Relative position of each stakeholder group will be assessed by comparing one group's position to other groups in broader scenario.
**Relative positions of**	Drivers	Stakeholders who have high level of importance as well as high level of influence on public sector health care delivery system

For peer review only - http://bmjopen.bmj.com/site/about/guidelines.xhtml

**stakeholders**	Supporters	Stakeholders who have high level of importance but low level of influence on urban healthcare delivery system
	Bystanders	Stakeholders who have low level of importance and low level of influence on urban healthcare delivery system
	Abstainers	Stakeholders who have no influence and no importance on urban healthcare delivery system
	Blockers	Stakeholders who have low level of importance but high level of influence on urban healthcare care delivery system.

For the *policy engagement analysis,* KIIs with stakeholders will be examined to understand interactions between actors, content, context and processes with respect to ICT policy uptake, with a view to identifying entry points for policy advocacy and the sustainable incorporation of ICT in health systems. Interviews will be analyzed using a priori codes drawn from the Policy Engagement Framework. Of additional interest in this analysis is understanding the mechanisms and processes of health policy development and how other policies may be important in efforts to integrate ICT into health systems. Based on these insights, a Health Policy for ICT process flowchart will be prepared. According to the policy engagement framework we will also seek information on how context is considered and dealt with when policies are formed and what processes need to be changed to more effectively integrate UHA into the system.[22]

For peer review only - http://bmjopen.bmj.com/site/about/guidelines.xhtml

For *quantitative data*, simple descriptive analysis will be performed to show user rates over time. The set of parameters to be analyzed are: number of users who accessed UHA, type of user, scope of UHA use, and types of problems faced. For data analysis, software like MS Excel and STATA will be used as appropriate.

The *cost* of implementing UHA will be estimated using a direct approach. Average cost for each activity will be calculated. All supply-side inputs will be identified, quantified, and valued through record review and KIIs. Shared cost items, including salary, buildings, furniture, supervision, transportation, and vehicles will be identified through observation and interviews of relevant personnel. By considering the nature of inputs, these will be categorized into capital, recurrent as well as fixed and variable cost items. Shared costs will be apportioned by proportion of the time-involvement of the relevant items (office rent, common vehicle) to different activities. Inputs will be identified using discussion with relevant personnel, observation, and record review. If unavailable, market prices will be applied to estimate costs. The capital items will be annualized, and common costs will be apportioned as per requirement. Annual values of capital items will be estimated from their expected useful life years and annuitization will be done using 3% discounting rate whenever applicable.[34] The study will allocate the cost for shared items (e.g. office space, appliances) by using actual utilization of items for this activity. Utilization information for shared items will be collected from responsible project staff.

Finally, the cost for shared items will be estimated by multiplying percentage-use information with the total cost of the items. Total cost will be calculated by summing up the start-up and

For peer review only

implementation costs. Relative contribution of start-up and implementation cost will be calculated. The cost-drivers in each activity will be identified considering the larger share of total cost.

Process documentation

In addition to all these activities, the study investigators will systematically document implementation processes for policy uptake and institutionalization of UHA, focusing particularly on contextual factors and their influence on implementation using a process documentation template. Process documentation of this "pilot phase" of UHA institutionalization will generate supporting knowledge to be applied the phases of scale-up and sustain, as specified in the WHO/PATH toolkit.

Patient and public involvement

There will be no direct patient or public involvement in this implementation research. However, a Technical Advisory Group (TAG) will be formulated for project governance, which will consist of representatives from Government, development partners, NGOs, academicians and senior researchers and urban health actors. Regular meetings will be held with partners and staff for problem solving. At the end of the study, the TAG will comment on the study findings and contribute to the dissemination plan.

Ethics and Dissemination

The institutional review board of icddr,b is comprised of two committees: the Research Review Committee (RRC) and Ethical Review Committee (ERC). This study has received approval from

For peer review only - http://bmjopen.bmj.com/site/about/guidelines.xhtml

both of the committees, which provided a thorough and critical review of the protocol's technical and ethical aspects. Participants will be asked for written consent prior interviewing and will remain anonymous and unidentifiable. Tape recorders will be used to record discussions bur only after obtaining consent. All other form of data will be kept in locked storage, or controlled access folders, allowing only investigators of the study and members of the ERC of icddr,b to access information, if needed.

Findings from this research will be disseminated at various levels to develop interest and support from a wide variety of audiences i.e. public, private, NGO, civil society, and donors. In doing so, we hope to build a diverse constituency of individuals and organizations willing and able to translate evidence yielded by the study, into policy action.

Local dissemination: Findings will be presented to relevant local administrators, development partners and NGOs and other relevant parties (local health practitioners), researchers.

National dissemination: A series of interactive workshops and briefing sessions with various stakeholders will be arranged to create linkages with national fora. The main aim will be to translate findings in a more visual and engaging formats, i.e. research briefs and interactive project brochures, to reach a range of stakeholders.

International dissemination: This will include publishing findings in peer-reviewed journals and presenting in scientific forums, conferences, and symposiums, and linking with international learning platforms. The main objective is to contribute to global knowledge about context

For peer review only - http://bmjopen.bmj.com/site/about/guidelines.xhtml

specific strategies to incorporate ICTs into health systems, and challenges that must be anticipated for policy uptake necessary to introduce, scale up and sustain MNCH related ICTs in similar LMICs.

For peer review only

For peer review only - http://bmjopen.bmj.com/site/about/guidelines.xhtml

REFERENCES

1. Adams AM, Ahmed SM, Evans TG. Universal health care in Bangladesh–promises and perils. *Lancet Glob Health* 2018;6:e10-11.
2. Ahmed SM, Islam FK, Bhuiya A, ed. *Bangladesh Health Watch Report 2014: Urban Health Scenario: Looking Beyond 2015.* Dhaka: James P Grant School of Public Health, BRAC University, 2015.
3. Chowdhury AMR, Bhuiya A, Chowdhury ME, *et al*. The Bangladesh paradox: exceptional health achievement despite economic poverty. *Lancet* 2013;382:1734-45.
4. National Institute of Population Research and Training. *Bangladesh Urban Health Survey 2013; Final Report*. Dhaka: 4. National Institute of Population Research and Training, 2013.
5. National Institute of Population Research and Training. *Bangladesh Demographic and Health Survey 2014: Key Indicators*. Dhaka: 4. National Institute of Population Research and Training, 2016.
6. United Nations. Department of Economic and Social Affairs Population Dynamics.. World Urbanization Prospects2018. Available: https://esa.un.org/unpd/wup/publications/Files/WUP2018-KeyFacts.pdf
7. Alam SMN. Health service delivery: the state of government-non-government relations in Bangladesh. *Public Adm Dev* 2011;31:273-81.
8. Afsana K, Wahid SS. Health care for poor people in the urban slums of Bangladesh. *Lancet* 2013;382:2049-51.
9. Huque R, Barkat A, Sabina N. Public health expenditure: equity, efficacy and universal health coverage. *In*: Ahmed SM, Mahmud S, Evans TG, *et al*. ed. *Bangladesh Health*

For peer review only - http://bmjopen.bmj.com/site/about/guidelines.xhtml

Watch Report 2011: Moving towards Universal Health Coverage. Dhaka: James P Grant School of Public Health, BRAC University, 2012:25-32.

10. Osman F. Public health, urban governance and the poor in Bangladesh: policy and practice. *Asia-Pacific Dev J* 2009;16:27-58.
11. Matthews Z, Channon A, Neal S, Osrin D, Madise N, Stones W. Examining the "urban advantage" in maternal health care in developing countries. *PLoS Med* 2010;7:e1000327.
12. Local Government Division, Government of the People's Republic of Bangladesh. Urban Primary Health Care Services Delivery Project. Available: https://www.adb.org/projects/42177-024/main
13. Nutley T, Reynolds HW. Improving the Use of Health Data for Health System Strengthening. *Glob Health Action* 2013;6.
14. Few S. Data Visualization for Human Perception. In: *The Encyclopedia of Human – Computer Interaction*, 2nd Ed. Available: https://www.interaction-design.org/literature/book/the-encyclopedia-of-human-computer-interaction-2nd-ed/data-visualization-for-human-perception
15. World Health Organization. *Creating a Master Health Facility List*. Geneva: World Health Organization, 2013.
16. Rose-Wood A, Heard N, Thermidor R, *et al*. Development and use of a master health facility list: Haiti's experience during the 2010 earthquake response. *Glob Health Sci Pract* 2014;2:357-65.
17. World Health Organization. *Toolkit on monitoring health systems strengthening Measuring Health Systems Strengthening and Trends: A Toolkit for Countries*. Geneva: World Health Organization, 2008. Available at:

For peer review only

For peer review only - http://bmjopen.bmj.com/site/about/guidelines.xhtml

https://www.who.int/healthinfo/statistics/toolkit_hss/EN_PDF_Toolkit_HSS_Introduction.pdf

18. World Health Organization/PATH. *Planning an Information Systems Project: A Toolkit for Public Health Managers*. Seattle: PATH 2013.
19. World Health Organization. Master facility list resource package: guidance for countries wanting to strengthen their master facility list. Available at: https://www.who.int/healthinfo/country_monitoring_evaluation/mfl/en/
20. Adams AM, Ahmed S., Hasan SM,. *et al*. *Mapping the Urban Healthcare Landscape in 5 City Corporations, Bangladesh*. Dhaka: icddr,b, 2015.
21. icddrb. The Urban Health Atlas. Available: http://www.urbanhealthatlas.com
22. Buse K, Booth D, Murindwa G, *et al*. *Donors and the Political Dimensions of Health Sector Reform: The Cases of Tanzania and Uganda*. London: Overseas Development Institute, 2008.
23. Peters DH, Adam T, Alonge O, Agyepong IA, Tran N. Implementation research: what it is and how to do it. *BMJ* 2013;347:f6753.
24. Archangel N. *The critical issues affecting the introduction of Health Management Information Systems in developing countries in Africa*. Amsterdam: Universiteit van Amsterdam, 2007.
25. Kilbourne AM, Neumann MS, Pincus HA, Bauer MS, Stall R. Implementing evidence-based interventions in health care: application of the replicating effective programs framework. *Implement Sci.* 2007;2:42.
26. Guest G, Bunce A, Johnson L. How many interviews are enough? An experiment with data saturation and variability. *Field Methods.* 2006;18:59-82.

For peer review only

27. Crouch M, McKenzie H. The logic of small samples in interview-based qualitative research. *Soc Sci Inf.* 2006;45:483-99.

28. Bryson JM. What to do when stakeholders matter: stakeholder identification and analysis techniques. *Public Manag Rev*. 2004;6:21-53.

29. Bryson JM. *Strategic planning for public and nonprofit organizations: A guide to strengthening and sustaining organizational achievementVol 1.* San Francisco, CA: Jossey-Bass Publishers, 1995.

30. Moore MH. *Creating public value: Strategic management in government*. Cambridge, MA: Harvard University Press, 1995.

31. Gold MR, Siegel JE, Russell LB, *et al*. ed. *Cost-effectiveness in Health and Medicine 1ed*. New York: Oxford University Press, 1996.

32. Drummond MF, Sculpher MJ, Claxton K, *et al*. *Methods for the economic evaluation of health care programmes*. Oxford: Oxford University Press 2015.

33. World Health Organization. *Comprehensive Multi-Year Planning (cMYP): A Tool and User Guide for cMYP Costing and Financing*. Geneva:World Health Organization, 2014.

34. Fiedler JL, Villalobos CA, De Mattos AC. An activity-based cost analysis of the Honduras community-based, integrated child care (AIN-C) programme. *Health Policy Plan* 2008;23:408-27.

For peer review only - http://bmjopen.bmj.com/site/about/guidelines.xhtml

Figure 1. Modified conceptual framework for Information Communication and Technology (ICT) implementation in developing countries

For peer review only

For peer review only - http://bmjopen.bmj.com/site/about/guidelines.xhtml

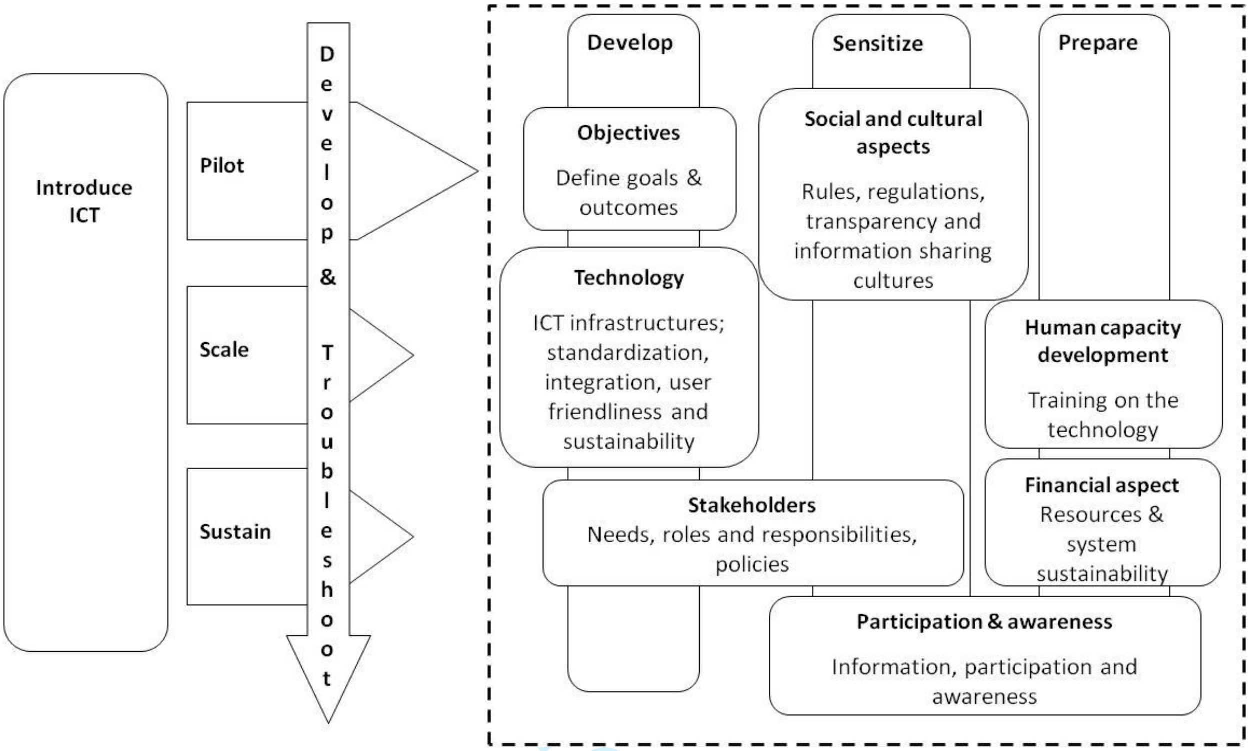

Figure 1. Modified Conceptual framework for Information Communication and Technology (ICT) implementation in developing countries

For peer review only - http://bmjopen.bmj.com/site/about/guidelines.xhtml

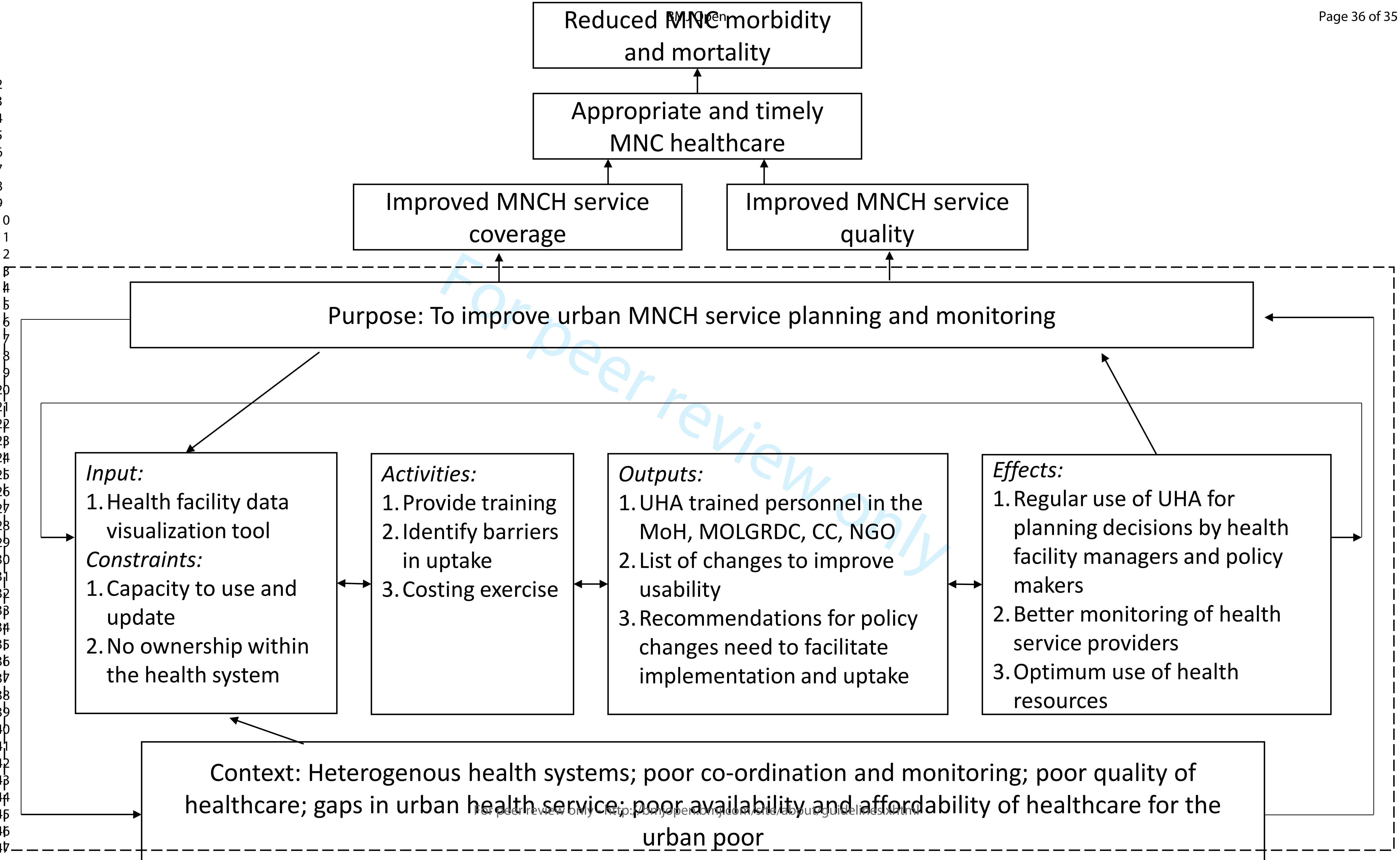

For peer review only
